# Mitigation and detection of putative microbial contaminant reads from long-read metagenomic datasets

**DOI:** 10.1099/mgen.0.001609

**Published:** 2026-01-22

**Authors:** Stefany Ayala-Montaño, Ayorinde O. Afolayan, Raisa Kociurzynski, Ulrike Loeber, Sandra Reuter

**Affiliations:** 1 Institute for Infection Prevention and Control, Medical Centre – University of Freiburg, Freiburg, Germany; 2 Max Delbrück Center for Molecular Medicine in the Helmholtz Association, Berlin, Germany; 3 Charité – Universitätsmedizin Berlin, Berlin, Germany; 4 Experimental and Clinical Research Center (ECRC), a cooperation between the Max‐Delbrück‐Center for Molecular Medicine in the Helmholtz Association and the Charité, Berlin, Germany; 5 German Centre for Cardiovascular Research (DZHK), Partner Site Berlin, Berlin, Germany

**Keywords:** contamination, *Enterobacterales*, genomic surveillance, long-read sequencing, metagenomes, nanopore

## Abstract

Metagenomic sequencing of clinical samples has significantly enhanced our understanding of microbial communities. However, microbial contamination and host-derived DNA remain a major obstacle to accurate data interpretation. Here, we present a methodology called ‘Stop-Check-Go’ for detecting and mitigating contaminants in metagenomic datasets obtained from neonatal patient samples (nasal and rectal swabs). This method incorporates laboratory and bioinformatics work combining a prevalence method, coverage estimation and microbiological reports. We compared the ‘Stop-Check-Go’ decontamination system with other published decontamination tools and commonly found poor performance in decontaminating microbiologically negative patients (false positives). We emphasize that host DNA decreased by an average of 76% per sample using a lysis method and was further reduced during post-sequencing analysis. Microbial species were classified as putative contaminants and assigned to ‘Stop’ in nearly 60% of the dataset. The ‘Stop-Check-Go’ system was developed to address the specific need of decontaminating low-biomass samples, where existing tools primarily designed for short-read metagenomic data showed limited performance.

**Impact Statement**

Metagenomics has gained popularity due to its diverse applications in the multi-omics research field and the improvements in sequencing performance of technologies such as Nanopore. However, challenges in biological interpretation remain because of the complexity of the data structure and the potential of contamination occurring at multiple steps during sample processing, which can lead to incorrect conclusions. We aim to raise awareness of contamination, which can be host-associated, cross-contamination or library-derived, any of which may be introduced at any stage from sample collection. Existing decontamination tools are largely designed for short-read sequencing and thus present limitations when applied to long-read datasets. We propose a direct comparison of species in samples with species in weekly negative controls that progressively accumulate both external and kit-reagent contaminants. Additionally, we recommend incorporating read-depth coverage and read-prevalence metrics, particularly in studies involving low-biomass or non-culturable micro-organisms. Whenever possible, validation with microbiological reports is strongly advised. Our code is available on GitHub and can be executed locally in RStudio. It outputs species classifications labelled ‘Stop’, ‘Check’ or ‘Go’, as well as BIOM format files clean of identified contaminants, ready for downstream analysis with R packages such as phyloseq, vegan or metagenomeSeq.

## Data Summary

The complete source code and documentation are available from GitHub (https://github.com/SAM81221/Stop-Check-Go_TAPIR). Metagenomic sequences, including controls, have been deposited in the ENA in project PRJEB82667, and isolate sequences of control samples in PRJEB95992. Information on samples and sequences can be found in Table S1, available in the online Supplementary Material 1.

## Introduction

Metagenomics has established a framework for better understanding the composition and interactions within microbial communities, shedding light on clinical applications, metabolic functional interactions and the long-term role of microbes in human health and disease. Traditionally, microbiome research relied on 16S rRNA gene sequencing, but this approach has limitations in resolving lower-order taxonomic classifications, and additional discriminatory power is required [1]. With advancements in sequencing technologies using high-throughput platforms such as Illumina technology and now Oxford Nanopore Technology (ONT), researchers can achieve strain resolution and gain more comprehensive insights into microbial dynamics and diversity. However, as the field has progressed, it is becoming increasingly evident that contamination remains a challenge, particularly for low-biomass samples, such as those involving neonatal samples or other clinical specimens with limited microbial content, where external and internal contaminants can skew results and significantly impact the accuracy and reliability of data interpretation [2]. Contaminants can originate from external sources such as laboratory surfaces, reagents, sample handling and instruments/equipment, or internal sources, including cross-contamination, arising during sample processing or index switching/hopping in sequencing [3,4]. These contaminants are particularly problematic in studies targeting specific taxa, such as Enterobacterales, as their presence may be falsely attributed to biological significance. For instance, in transmission studies, contamination could lead to erroneous conclusions about microbial transmission events. Similarly, in microbiome profiling/metataxonomic assignment, contaminants can shift taxonomic profiles, potentially masking true microbial signals and biassing ecological interpretations. While these issues may be less relevant in high-biomass samples, such as soil, where contaminants are more readily distinguishable, their impact in clinical and low-biomass studies necessitates stringent measures for identification and mitigation [5].

Recently, a comprehensive guidance for preventing contamination has been published for the design of sample collection and processing in metagenomic studies with strategies to minimize contamination and cross-contamination that aim to provide standard guidelines [6]. In this guide, there are approaches (moderate to essential relevance) in areas such as awareness/training, sample collection, laboratory practices and data analysis/reporting. One key message is to ensure the awareness of contaminants introduced at any point from sample collection to sequencing by continuously providing training and audits in the processes. This awareness leads to the second key message, which is the inclusion of negative controls that progressively ‘accumulate’ the contaminants during the sample processing [6], which we believe is a major limitation in this field. Studies have shown that nearly 70% of metagenomic studies do not include negative and technical controls, and of those that do include them, only a fraction sequence and analyse them [7]. This oversight complicates the ability to distinguish true microbial signals from contaminant DNA. Additionally, certain contaminant taxa – over 60 of which have been identified in the literature – are context-dependent, varying based on the specific kits and laboratory environments used. These taxa collectively shape the ‘contabiome’ [8], and their acknowledgement is crucial for interpreting results and making informed decisions in microbiome research [8]. Here, it is useful to store and report reagent production changes and, if possible, the location of the samples on the well plate to keep track of batches changing, which can be useful to determine the nature and the extent of the introduced contamination. Contamination is challenging to identify as it involves micro-organisms also present in the samples, and regardless of the mitigation techniques such as UV irradiation, purification or the separation of working areas, complete elimination is rarely achievable [3,7,9].

To address this issue, several tools have been developed for bacterial contaminant identification, including Decontam [7], SourceTracker [10], SCRuB [11] and MicrobIEM [12]. These tools employ different methodologies to differentiate true signals from contaminants, relying on sequencing data from negative or environmental controls [8]. Decontam uses frequency-based methods (which assume that contaminant DNA is inversely correlated with input DNA) or prevalence-based methods (which compare the presence of taxa in negative controls versus biological samples) [7,13]. SourceTracker and SCRuB employ a Bayesian model to estimate the proportion of n sequences in a defined sink sample x by using the Gibbs distribution [10] and form part of the open-access QIIME tools for metagenome and 16S rRNA gene amplicons [13]. MicrobIEM incorporates the prevalence method of Decontam and provides options based on the ratio of the species mean relative abundance, which requires environmental sampling [12].

In this study, we investigated contamination in metagenomic samples obtained from low-biomass screening samples collected weekly from premature and term neonates in the neonatal intensive care unit (NICU) at the Medical Centre – University of Freiburg. These samples were analysed using ONT long-read sequencing and presented challenges, as low-biomass samples are particularly prone to contamination due to their low microbial load, high host DNA content and susceptibility to technical artefacts [14,15]. To address these challenges, we developed a system called ‘Stop-Check-Go’ by adopting the prevalence method in Decontam [7] and incorporating a novel metric – coverage per species per sample. This system leverages the advantages of long-read sequencing, which enables more comprehensive genome and metagenome characterization, providing critical insights into microbial diversity and its implications for health and disease [13,16].

Through this approach, we not only accounted for contamination but also highlighted the importance of implementing negative controls and integrating them into metagenomic analyses to enhance the reliability of results. By addressing contamination systematically, we aim to improve the accuracy of strain-level microbial profiling, particularly for Enterobacterales, to understand microbial transmission and microbiome dynamics in neonatal health [15,17].

## Methods

### Collection of samples, microbial growth and preservation

Rectal and nasal swab samples were routinely collected from neonates admitted to the NICU within the framework of a study investigating the emergence, spread and dynamics of healthcare-associated bacteria with the intent of tracking the acquisition of pathogens in real-time (TAPIR). We included sterile swabs and molecular-grade water as weekly negative controls, which were processed and sequenced alongside the biological samples. All samples were enriched overnight in Brain Heart Infusion (BHI) broth at a 1 : 2 ratio of sample to media at 37 °C, 210 r.p.m., to select for Enterobacterales, our focus group of organisms. Microbiological reports were received from the diagnostics department, and we validate these results by culturing on MacConkey agar, CHROMagar^™^/CNA agar (biplate) and blood agar, incubated at 37 °C for 24 h. Positive-growth samples on MacConkey agar (associated with Enterobacterales) were identified using MALDI-TOF. These cultures were sequenced (details in the ‘Removal of human DNA and DNA extraction’ section) and were available in cases where confirming true positives was needed. Sweeps of samples with Enterobacterales and purified bacteria were cryopreserved in horse blood at −80 °C.

### Removal of human DNA and DNA extraction

Human DNA (hDNA) was removed from enriched broth samples by centrifugation at 10,000 r.p.m. for 5 min, and the pellet was incubated with 1 ml of molecular-grade water at room temperature for 5 min, promoting eukaryotic cellular lysis [18]. Samples were centrifuged at 10,000 r.p.m. for 5 min before resuspension of the microbial pellet in PBS 1X. A ratio of 1 : 1 lysozyme+lysostaphin was used for DNA extraction using the kit High Pure PCR Template (Roche Molecular Diagnostics, Mannheim, Germany) following the manufacturer’s protocol (catalogue no. 11796828001). DNA libraries for pure cultured isolates were prepared with the Nextera DNA Flex Library Preparation Kit (Illumina). Paired-end sequencing (2×150 reads, 300 cycles v2) was conducted on an Illumina MiSeq platform.

### Library preparation and metagenomic sequencing

On average, we collected 24 samples per week, corresponding to 12 patients with two sets of samples, which were processed and distributed on two identical flow cells. Libraries for long-read sequencing were prepared using the ligation sequencing kit (SQL-LSK109; ONT, Oxford, UK) and native barcoding kits (EXP-NBD104 and EXP-NBD114) for R9 Chemistry (from the beginning of the project until week 13). The SQK-NBD114-96 barcoding kit for R10 chemistry was used from week 14, following the Ligation sequencing gDNA - Native Barcoding Kit 96 V14 protocol (NBE_9171_v114_revF_15Sep2022). In the R10 chemistry, we used an initial volume of 11 µl per sample in the library protocol to get the maximum possible concentration (low-biomass samples ranged from too low to 3.5 ng µl^−1^ after BHI enrichment). Final libraries were sequenced on a GridION X5 Mk1 sequencing platform. Sequence data acquisition, real-time base-calling and demultiplexing of barcodes were conducted using the graphical user interface MinKNOW (v23.11.7) and Dorado basecall server (v7.2.13). From week 17, Readfish (v23.1.1) was run simultaneously with the start of the sequencing to actively target and ‘reject’ sequencing reads corresponding to hDNA (GRCh38, T2T-CHM13v2) across individual nanopores [19]. Weeks with a final library concentration below 7.0 ng µl^−1^ and negative growth in the controls were repeated with R10 chemistry flow cells. The 11 µl loading volume per sample was chosen for a good initial concentration and to minimize the risk of contamination based on the prevalence method (refer to results section: Implementation of the Stop-Check-Go system).

### Read pre-processing and removal of hDNA contaminants

Sequenced data underwent pre-processing using Porechop (v0.2.4; https://github.com/rrwick/Porechop) to trim off adapters. Trimmed reads were filtered using Filtlong (v0.2.1; https://github.com/rrwick/Filtlong) with a minimum read length threshold (‘--min_length’) of 1,000. Afterwards, reads were mapped to the human genome (GRCh38, T2T-CHM13v2) using Minimap2 (v2.24; using the ‘map-ont’ parameter) [20], and hDNA reads were removed using SAMtools (v1.14) [21]. These steps, including read trimming, read filtration and hDNA removal, were executed using custom Nextflow pipelines [22,23] (see Table S2 in Supplementary Material 1 for the codes implemented in Nextflow pipelines). Metagenomic sequences, including controls, have been deposited in the ENA in project PRJEB82667. Information on samples can be found in Table S1 (Supplementary Material 1).

### Microbial species abundance estimation of real-world metagenomic datasets

Metagenomic composition reports were generated using Kraken v1.1.1 [24] with the MiniKraken database (DB_8 GB), and the microbial abundance was estimated from this output using Bracken v2.8 [25]. The taxonomic profiles per sample per week were visualized in R, using Tidyverse (v2.0.0) [26], scales [27] and ggthemes [28] to validate the microbiological reports. Those controls associated with positive growth were purified, confirmed by MALDI-TOF, sequenced with the Nextera DNA Flex Library Preparation Kit (paired-end sequencing, 2×150 reads, 300 cycles v2, Illumina MiSeq), and the species were identified to the sequence type level using MLST [29,30]. We labelled the contaminants in the Kraken metagenomic reports and excluded them from downstream analyses if the same sequence type for the corresponding week was identified with TRACS [31]. These sequences have been deposited in the ENA in project PRJEB95992.

### Stop-Check-Go system development and identification of non-hDNA contaminants

The Stop-Check-Go system was designed to include two main quantitative parameters and one qualitative parameter: Difference Fraction Reads (DFR), coverage (read depth) and the presence of microbial contaminants in negative controls. Individual taxonomic reports were combined and used to calculate the (1) difference of the fraction reads (DFR) per species per sample based on the prevalence method that included the reads fraction of the control (FRC) of species x, and the reads fraction in the sample (FRS), *DFR=FRC*−*FRS*. The (2) coverage values were obtained from the assembly statistics text file from FLYE [32] by the ‘--stats’ parameter for n contigs per sample file. We used the ‘Kraken-translate’ script [24] to obtain the taxonomic association within each contig and therefore could associate the ‘contig_n’ to a species. Binary microbiological growth results per sample (positive, negative), including controls, were added to the extensive combined Bracken Kraken file, which incorporated three parameters to classify and identify potential contaminants. The complete source code and documentation are available from GitHub (https://github.com/SAM81221/Stop-Check-Go_TAPIR).

### Comparison of the Stop-Check-Go with other decontamination tools

The ‘Stop-Check-Go’ system was compared across 5 weeks with decontamination tools Decontam [6] and MicrobIEM [12]. Each method generated a list of putative contaminants ‘tax_id’ that was used to decontaminate the dataset, where each ‘tax_id’ was located in the respective tables containing the observation counts for each taxonomic unit (otu_table, phyloseq object). A new otu_table was generated from all decontamination tools, where zero was assigned to every putative contaminant otu (in the corresponding positions). These decontamination outputs were normalized using the Normalization.R script [33], and Jensen–Shannon diversity (JSD) was calculated to quantify the communities’ species distribution. Non-parametric Spearman correlation and Mantel statistics were calculated for each paired combination of the decontamination tools.

## Results

### Collection overview

A total of 1,351 samples were collected from 302 patients, with 182 (60%) staying 1 week on the ward, 53 (18%) staying 2 weeks, 20 (7%) staying 3 weeks and 47 (15%) staying more than 3 weeks. Overall, 58% of the patients (*n*=177) were colonized by Enterobacterales, and 25 patients were found to be microbiologically negative and were discharged before a second screening. Two negative controls from sterile swabs and molecular-grade water were processed weekly, bringing the total to 1,475 samples analysed.

### hDNA removal

We evaluated the presence of hDNA contaminants in our samples as hDNA usually misleads the interpretation of the results [34,35]. We have applied three different approaches for the removal: previous to DNA extraction (pre-sequencing), during sequencing and post-sequencing. For pre-sequencing, we processed 289 samples (across 10 weeks) and compared different methods, ordered by the duration in the lab (for 20 samples): filtration, lysis, DNase, Molzym kit and saponin (35, 45, 55, 120 and 180 min, respectively). The samples were sequenced, and the reads mapped/unmapped to the hDNA are in Supplementary Material 1 Table S3, sheet: week39to49_bam_stats_and_metada. We selected the lysis protocol based on performance and time. We then processed 11 samples by dividing each into three equal-volume aliquots corresponding to three independent conditions: untreated–unenriched, unenriched–treated and enriched–treated (also provided in Supplementary Material 1 Table S3, sheet: three_conditions_tested). This enabled us to compare the effect of the treatment (lysis), which yielded a 70–90% in host DNA reduction, and an increase of 86.87% in microbial DNA.

During sequencing, we used a Readfish Python package (Supplementary Material 1 Table S2, A.) that selectively ejected reads from the flow cell pores matching the hDNA reference (GRCh38), decreasing the number of human reads. We evaluated the performance of Readfish for blocking and rejecting hDNA in 4 weeks with a total of 102 samples after 24, 48 and 72 h of sequencing. We could observe an increase in the number of microbial reads, including low-biomass samples, while the species composition remained largely unchanged. We provide an example of Readfish (adaptive) performance compared to normal sequencing at 72 h in Supplementary Material 2 Fig. S1A, and the species composition in Fig. S1B. We quantified that the content of hDNA in the samples was higher in the nose than the anus, and the proportion of matching hDNA reads across the weeks is provided in Supplementary Material 1 Table S4. The remnant hDNA and microbial DNA reads are presented in Supplementary Material 2 Fig. S2. In post-sequencing analyses, after Porechop and Filtlong (Supplementary Material 1 Table S2, B.), we filtered the reads that mapped against the reference GRCh38 and T2T-CHM13v2 with minimap2, from which we retained only the microbial DNA for downstream analyses using SAMtools (Supplementary Material 1 Table S4) [21].

### The residual number of reads in sample types might be associated with contamination

**Fig. 1.**
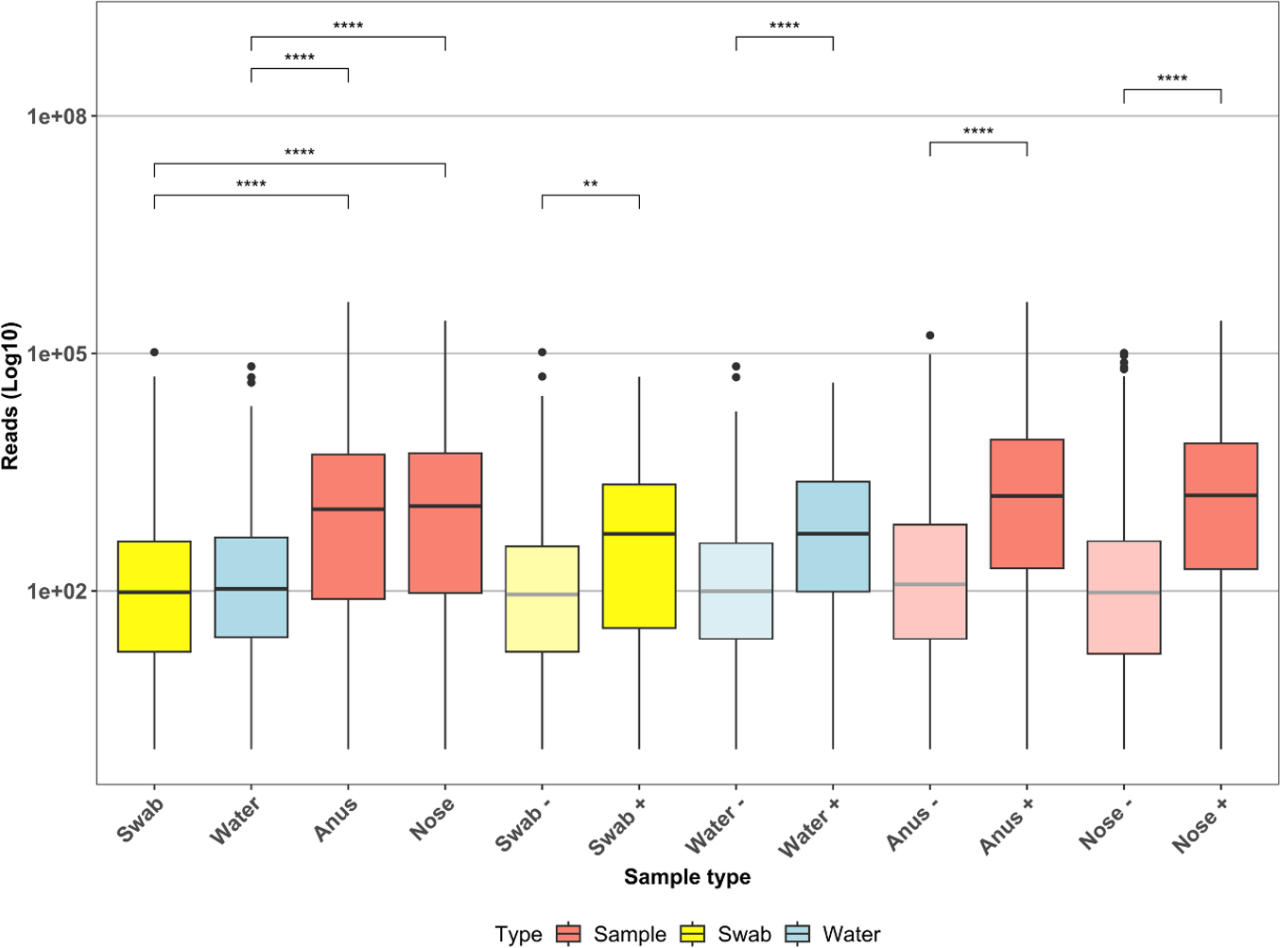
Average number of reads (log10) between the sample types: swab, water (controls), anus and nose (samples). Lighter shading indicates microbiologically negative samples. The Wilcoxon signed-rank test between all sample types (globally), the controls and the samples (only) (p. adj.<0.05).

We sequenced 25 microbiologically negative patients and 105 negative-growth controls (85%, *n*=124), and regardless of reported negative growth on agar plates, we quantified reads associated with the introduction of microbial contaminants. We found that most of the species in these samples had less than 1,000 (1K) reads; however, certain species contributed a large number of reads (>1K). *Staphylococcus epidermidis*, *Staphylococcus aureus*, *Klebsiella pneumoniae* and *Escherichia coli* contributed with reads between 1K and 10,000 (10K), and *S. epidermidis* exhibited reads between 10K and 100,000. We checked the weeks with reads in negative samples and found a homogeneous distribution across the dataset, although the biggest effect was attributable to weeks 22, 23 and 24 and 24, 25 and 40 (nose and anus, respectively). This elevated number of reads in the negative samples and controls led us to compare the read abundance among the sample types. We found significant differences (p-adj.) in the controls (swab, water) versus the samples (anus, nose) (Fig. 1). We further separated the sample types by positive or negative growth and showed that regardless of the high number of reads in the negative samples, the number in the positive samples is markedly higher with statistical significance (p-adj., Holm correction method).

### Contaminant detection and source attribution

**Fig. 2.**
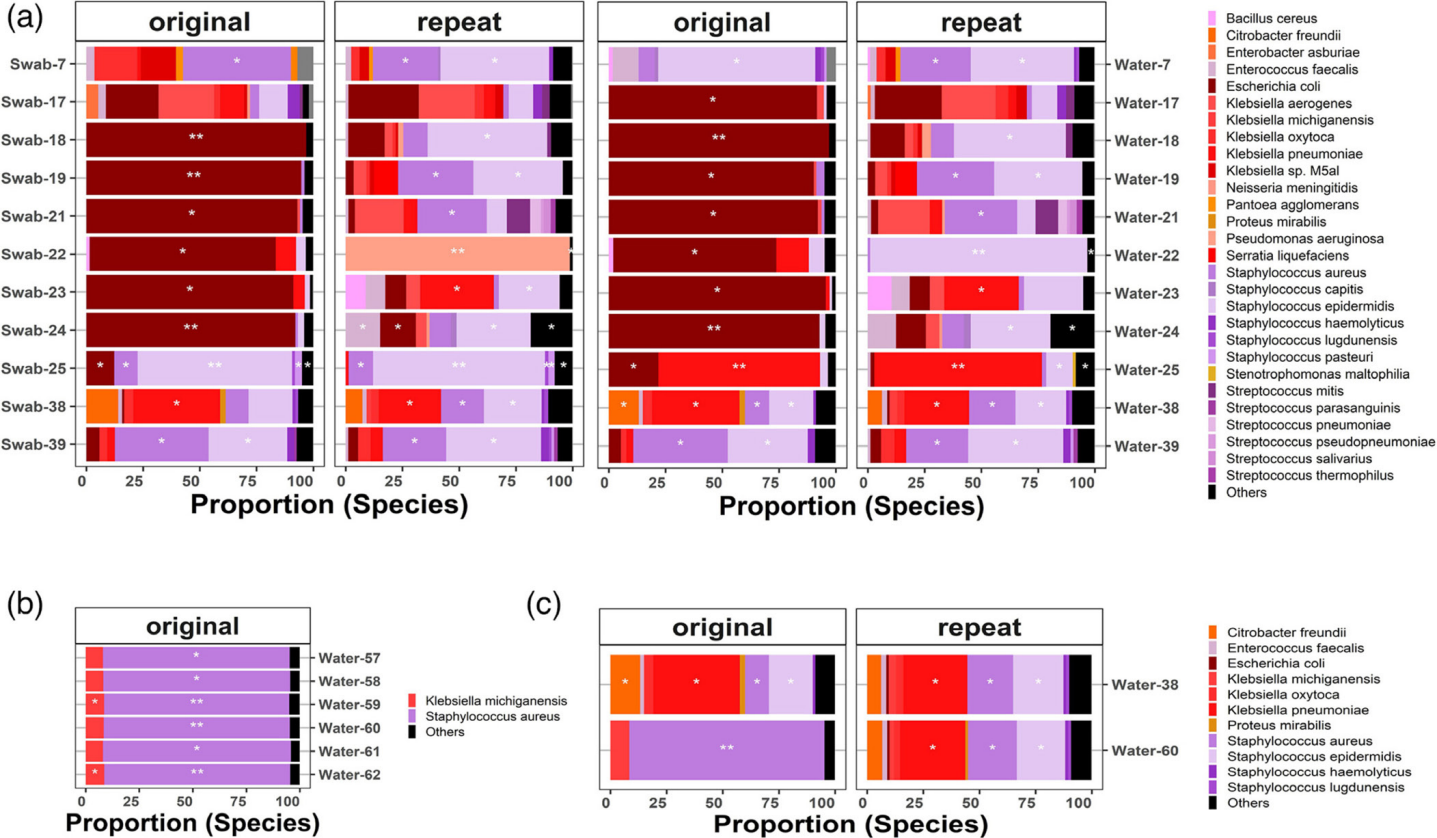
Taxonomic profile of chronologically ordered controls (swab and water). Species with less than 1,000 reads are denoted by no star. A single white star indicates species with 1,000 to 10,000 reads, while double white stars on stacked bars represent species with ≥10,000 reads assigned to them. Black represents other micro-organisms.

The high number of reads found in the negative-growth samples justified the need to distinguish the contamination sources and to develop a strategy for dealing with potential microbial contaminants. We identified three types of contaminants: (a) cross-contamination (between samples) either before or during the enrichment step, (b) inherent reagent contaminants during DNA extraction and library preparation and (c) additional contamination where the species appeared especially in low-biomass samples at higher abundance, now termed library contamination. The cross-contaminant species (a) were frequently observed growing on negative controls where sequencing of purified bacteria was done, and false positives could be ruled out using the microbiological reports. We reported 15 weeks with cross-contamination (24%), but only 3 weeks (5%) with Enterobacterales in swab/water: week 12 *E. coli* sequence type (ST) 8186 and weeks 15 and 16 *Klebsiella oxytoca* ST189. The contamination in (b) appeared in the samples, but, crucially, also in the negative controls in low proportions, and was not represented in any case by Enterobacterales. The profile of the contaminants in (c) will shift from a mixed-species profile to one where a single contaminant is highly abundant. We had such cases in our study, and we could demonstrate that this additional contamination was removed after library repeat (Fig. 2a).

**Fig. 3.**
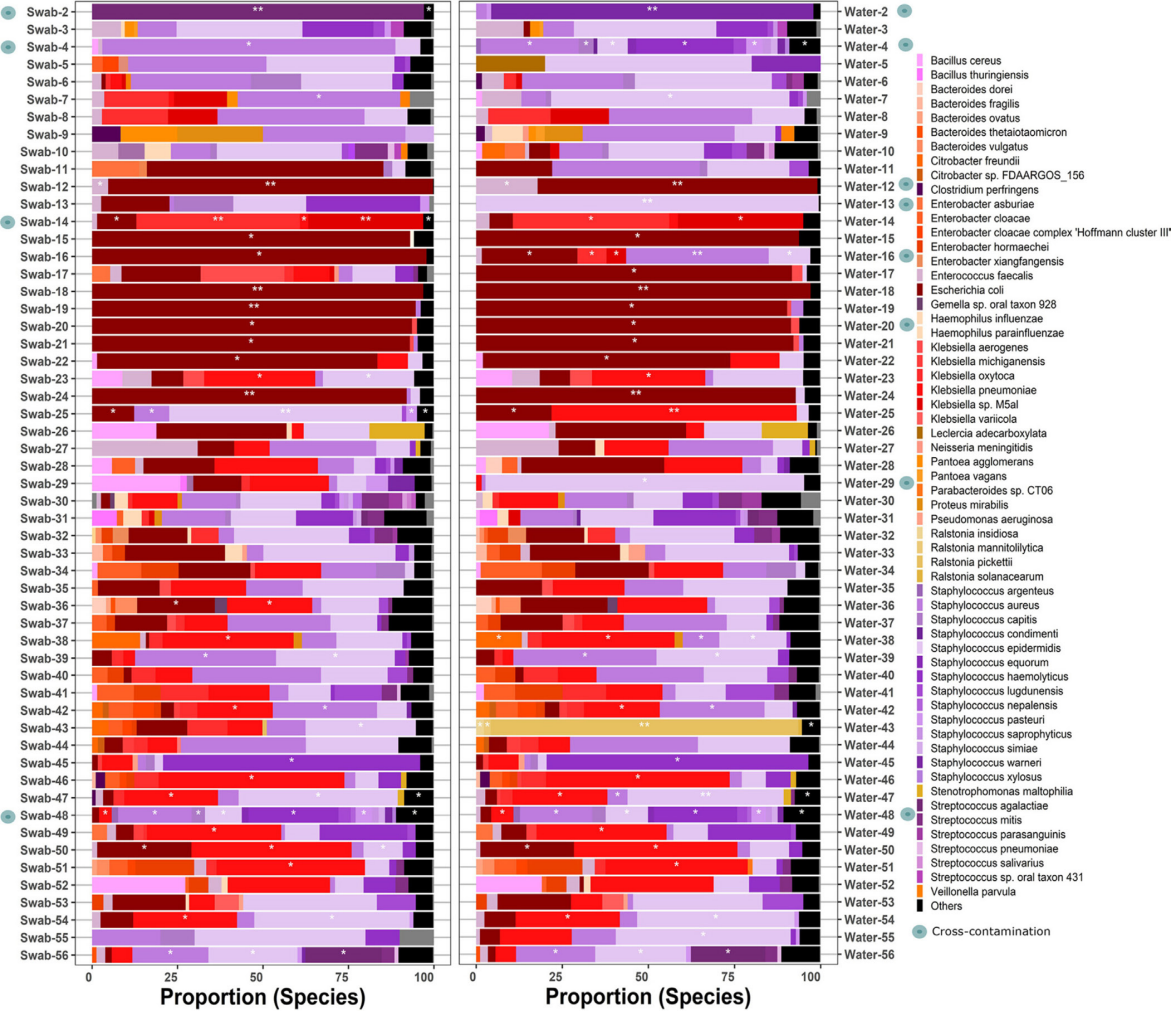
Taxonomic profiles of libraries with low-concentration libraries (week 7 to week 24) where library contamination was suspected. Libraries with good concentrations (weeks 25, 38 and 39) were used as controls. (a) Original and repeated libraries (swab and water). Species with less than 1,000 reads are denoted by no star. A single white star indicates species with 1,000 to 10,000 reads, while double white stars on stacked bars represent species with ≥10,000 reads assigned to them. Black represents other micro-organisms. Samples termed as repeats correspond to library repetitions with R10 flow cell chemistry. (b) Taxonomic profiles of the negative controls for the last patient in this study across 6 weeks. (c) Batch effect in the repeated sequencing libraries of different patient samples.

From the observation and quantification of negative controls, we can suspect the contamination status of samples and controls. We expected that samples with low or near-zero contamination would have a mixed taxonomic composition profile with a low number of reads (<1K, denoted as ‘no star’). We also expected the species proportion to show variation in the library preparation. Examples of low contamination profiles for the controls (regardless of the type: water or swab) are weeks 8–10, 30–35 and 52–53 (Fig. 3). The weeks 17–19 and 21–25 are possible examples of contamination (Fig. 2a), where controls in the weeks 18–24 are represented by the dominance of a single species, in this case, *E. coli*. Validation by culturing showed negative growth, and MLST from the metagenomic negative control samples (with reads above 1K and 10K) led to inconclusive results as the ST could not be identified.

### Library repetition improved the DNA concentration and taxonomic profiling

The weeks that did not follow the expected mixed pattern were further investigated, and we found that 70% of the libraries (*n*=7/10) had low library concentrations with low individual DNA extraction concentrations. In addition, we extracted the reads of the overrepresented *E. coli* in the controls, which had more than 10K reads (in most cases); however, we were unable to assign a particular ST to any of these *E. coli*. The combination of low DNA concentration, negative growth in the controls and unavailable STs led us to select 7 weeks with this particular library contamination for further examination. The library preparation for these weeks was repeated (including the controls) with the R10 chemistry. Table 1 summarizes the final library DNA concentration (before sequencing) of the originals and the repeats. We included five additional libraries with either low DNA concentration (week 7) or high concentration but the presence of Enterobacterales (>1K) and negative growth (weeks 25 and 38). Two additional libraries were included as negative controls (Enterobacterales<1K, weeks 39 and 60) for a total of 12 repeated libraries. There was a marked improvement in the library concentration in 75% of the repeated libraries, except for weeks 25, 38 and 39, which had good concentration in the original libraries (see Table 1). The taxonomic profiles of the original and repeated libraries are displayed in Fig. 2(a), where, in cases of *E. coli* overrepresentation, the controls of the repeated libraries reverted to mixed profiles. An exception occurred in week 22, where, after the repetition, we observed cross-contamination with *Pseudomonas aeruginosa* in the swab, which was not associated with positive growth during the culturing step.

**Table 1.** DNA concentration (in ng/µl) of the weeks in the first (original) and second (repeated) library preparation with R10 flow cells

Week	DNA concn original library	DNA concn repeated library (R10)	DNA concn improvement (%)
7	A: 22.66, B: 6.08	39.00	A: 172, B: 641
17	5.52	25.08	454
18	3.61	21.81	604
19	4.73	28.00	592
21	6.54	67.82	1037
22	5.02	A: 33, B: 6.0*	A: 657, B: 120
23	A: 2.15, B: 1.93	37.65	A: 1751, B: 1951
24	19.31	30.43	158
25†	56.6	14.15	−25
38†	63.01	30.83	−49
39†	72.0	57.6	−20
60	38.40	48.00	125

*Week 22 was split into two pools (A and B). In pool B, 3 out of 7 patients were microbiologically negative, which strongly diluted the final library concentration.

†Regardless of concentration, the libraries were repeated due to the suspicion of library contamination in the controls. A and B referred to Pool A and Pool B, respectively, which were loaded in different flow cells.

We closed the study in week 56 but followed the last patient until discharged as we reported colonization by *K. oxytoca* from week 54; however, due to the low number of samples (per week), we multiplexed all in a single library. This facilitated the identification of the strong batch effect in the libraries that were sequenced together as displayed in Fig. 2(b). In addition, the same effect was observed for week 60 (Fig. 2c) after being multiplexed with week 38, where the composition of the taxonomic profiles is nearly identical in both weeks.

### Classification of putative microbial contaminants in the ‘Stop-Check-Go’ system

The contaminants during DNA extraction, library preparation and sequencing that are difficult to identify and the observation of a high number of reads in the samples we presumed as negative, encouraged us to develop a classification system. This system called ‘Stop-Check-Go’ included microbiological reports, coverage and the weekly negative controls for validating the species presence with the prevalence method already published [7]. The species validation occurred by a direct comparison of the species in the samples with the species in the controls (by week) using the prevalence method proposed by Davis *et al.* [7]. The authors established that the total sample DNA (T) is composed of contaminant (C) and true sample DNA (S), so *T=C+S*. Thus, in negative controls where *S*~0, the probability of contaminant detection is higher than the detection of contaminants in true samples when *S*>0 [7]. This means that species present in the samples are classified as contaminants if species prevalence is higher in the controls: *Prevalence controls>Prevalence* *samples*. This follows the assumption that the species in the controls have competing DNA during the sequencing process, and therefore, it is a good portrait of species contaminants for the corresponding week [7]. We checked this prevalence by calculating the difference of the fraction reads (DFR) per species per sample. The values were classified as ‘Stop’ if *DFR=FRC* FRS>0, and ‘Go’ when *DFR*<0. Afterwards, we classified the species according to the read coverage after assembly and error correction (Flye, Medaka, respectively) with a minimum (condition 2, see Table 2) coverage of 20 as proposed by the literature [36]. We validated this chosen threshold using negative controls and microbiologically negative patients (MNPs) and found that 99% of these were distributed below 20X (Supplementary Material 2 Fig. S3).

**Table 2.** Summary of the decision for the Stop-Check-Go system that includes the parameters DFR, coverage and microbiological results

	Parameter 1	Parameter 2	Parameter 3 (auxiliary)	Outcome
Condition	Difference Fraction Reads (DFR)	Coverage	Contamination reason control	Decision system
(1)	Stop	Low	–	Stop
(2)	Stop	Ok	–	Stop
(3)	Go	Low	Library contamination 10K	Stop
(4)	–	–	Patient microbiologically negative	Stop
(5)	Go	Low	No reason	Check
(6)	Go	Low	Library contamination 1K	Check
(7)	Go	Ok	Cross-contamination	Check
(8)	Go	Ok	Library contamination 10K	Check
(9)	Go	Ok	No reason	Go
(10)	Go	Ok	Library contamination 1K	Go

Library contamination 10K refers to species in controls where the number of reads was higher than 10,000. ‘–’ denoted parameters not evaluated in the conditions.

Using this coverage, the species were classified as ‘low’ for coverage <20X, and ‘ok’ for coverage of 20X or above, and we complemented this with an auxiliary third parameter from microbiological reports. These reports were useful to classify the contamination reason of the controls, either cross-contamination (before DNA extraction) or library contamination (1K or 10K).

These three parameters were used to formulate the conditions of the classification system using the function ‘case_when’ from the dplyr R package, and each condition was evaluated independently. Table 2 summarizes the system decision parameters and the classification into the corresponding category: ‘Stop’, ‘Check’ or ‘Go’. Overall, the classification system strictly excluded species with a lower proportion of reads compared to the species in the negative controls *DFR*>0 (conditions 1 and 2), and microbiologically negative samples (condition 4), as the reads might arise from previous steps where the contamination was present. Species with a high number of reads in the negative controls (library contamination 10K) were considered as putative contaminants if the coverage was low (condition 3). Species in the ‘Check’ category (conditions 5–8) were examined individually with the microbiological reports, and only Enterobacterales found in pure culture were classified as ‘Go’. Three weeks (5%) were contaminated with Enterobacterales: *K. oxytoca* ST186 (weeks 15 and 16) and *E. coli* ST8186 (week 12). The other non-Enterobacterales contaminant species (*Staphylococcus* sp. and *Enterococcus* sp.) were classified to ‘Stop’.

### Dynamic classification of species across samples

**Fig. 4.**
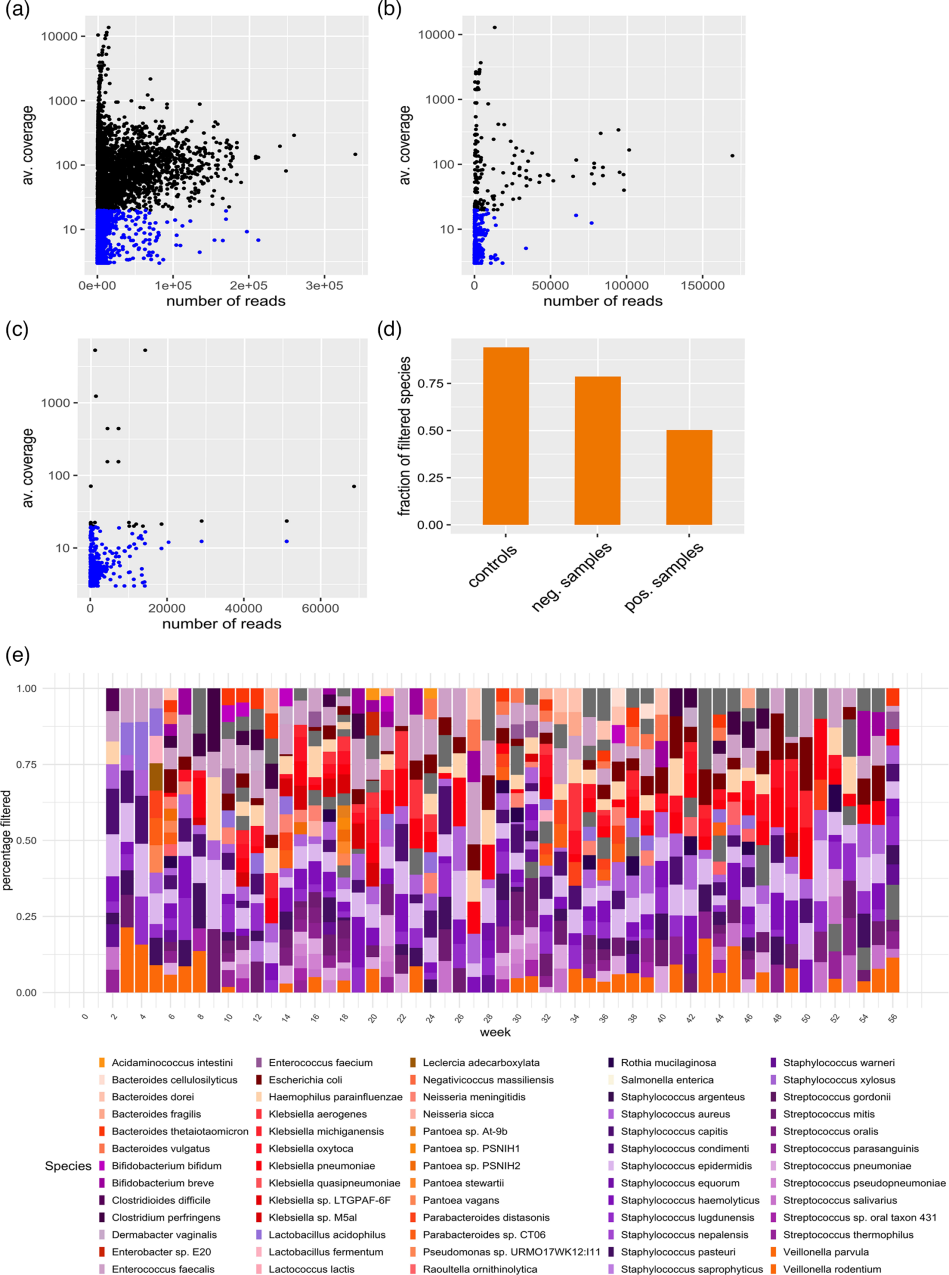
Average coverage per species in the metagenome versus the total number of reads. The blue dots indicate species with coverage below the 20-threshold. (a) The coverage for microbiologically positive samples. (b) Coverage for microbiologically negative samples. (c) Controls. (d) Fraction of filtered species based only on coverage. (e) Taxonomic profile of the filtered species based only on coverage.

Defining classification thresholds for putative contaminants based on read counts is a common approach in metagenome and microbiome studies [4,36, 37], but the use of coverage has the advantage of accounting for the genome size and read length. Consequently, species with a small genome size (e.g. *Staphylococcus* sp.) can be falsely filtered when using read counts as they have a small number of reads but still good coverage. We explored the relationship between the coverage and the number of reads within the metagenomes by classifying the samples based on microbiological reports for samples that were microbiologically positive (Fig. 4a), microbiologically negative (Fig. 4b) as well as the controls (Fig. 4c), and also reported the proportion of filtered species solely based on a coverage below 20X (Fig. 4d). The differences in read quality can be observed by the broad distribution of the dots in Fig. 4(a), and it is clear that even in negative samples, there are reads that can be misclassified based only on coverage (Fig. 4b). In the controls, we determined that all the samples, reported as microbiologically negative, were under the threshold of 20X. However, using only the coverage will create false positives in the samples where the controls had a high number of reads and good coverage (Fig. 4c), commonly observed in cross-contamination or library contamination 10K. On average, 50% of the species present in the metagenomes would be removed from the positive samples (Fig. 4d) without the inclusion of additional parameters, and ~25% of the microbiologically negative samples could not be filtered. The profiles and proportion of putative contaminants filtered using the defined coverage cutoff of 20 are presented in Fig. 4(e), where the species profile changed weekly. However, a common set of *Staphylococcus* sp., *Streptococcus* sp. and *Veillonella* sp. had the highest removal rate independently of the week.

**Fig. 5.**
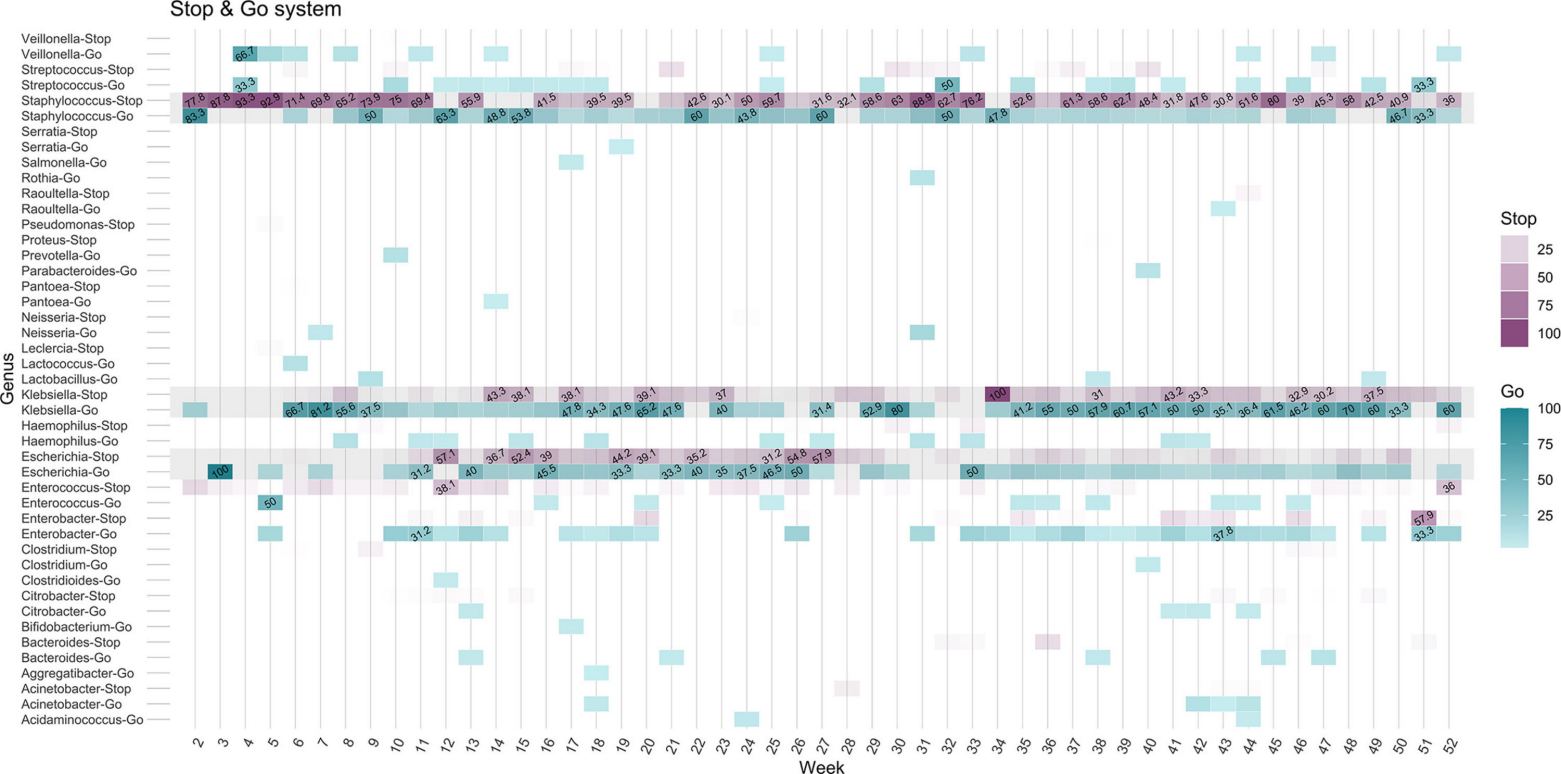
Overview of genus classified as ‘Go’ and ‘Stop’ within the system, showing the relative frequency of bacterial genera in the collected samples. Darker regions represent higher relative abundances, while values below 0.3 are not labelled.

When we applied the classification system ‘Stop-Check-Go’ to the dataset, the species that fell into conditions 1, 2, 3 and 4 from Table 2 were classified to ‘Stop’ and species into conditions 9 and 10 to ‘Go’. The overview of genus relative frequency per week is presented in Fig. 5, where 20 species were classified simultaneously in both categories (57.14%, total species=35). *Staphylococcus* sp., *Klebsiella* sp. and *E. coli* contributed to the highest rejection rates (‘Stop’) and inclusion rates (‘Go’) across the dataset. The detailed overview of the main genus contributing to high ‘Stop’ and ‘Go’ categories at the species level is shown in Supplementary Material 2 Fig. S4, where *S. aureus* and *S. epidermidis* had strong rejection rates and were therefore shown as contaminants in the study (Supplementary Material 2 Fig. S4).

The same analysis was done for the Enterobacterales; we found a higher average relative frequency of *E. coli* in the category to ‘Stop’ in weeks with suspected library contamination compared to the rest of the dataset (32.82% and 10.20%, respectively). Similarly, *K. pneumoniae* was observed with a higher frequency in ‘Stop’ and ‘Go’ in the second part of the study (Supplementary Material 2 Fig. S4); however, the effect on library contamination was underestimated as the dominance in the controls was not as complete as that of *E. coli*. The abundance of other *Klebsiella* sp. can be observed in Supplementary Material 2 Fig. S4, with *K. oxytoca* being more abundant than *Klebsiella aerogenes* and *Klebsiella michiganensis*. Overall, the Enterobacterales in ‘Check’ were compared with the microbiological reports to determine the proportion that could be confirmed to ‘Go’. A total of 17.45% (204, total=1,169 samples in ‘Go’) were confirmed as positive by microbiology for *Klebsiella* sp. (*K. aerogenes*: 4, *K. oxytoca*: 22, *K. pneumoniae*: 97, *Klebsiella variicola*: 2) and *E. coli* (*n*=79).

**Fig. 6.**
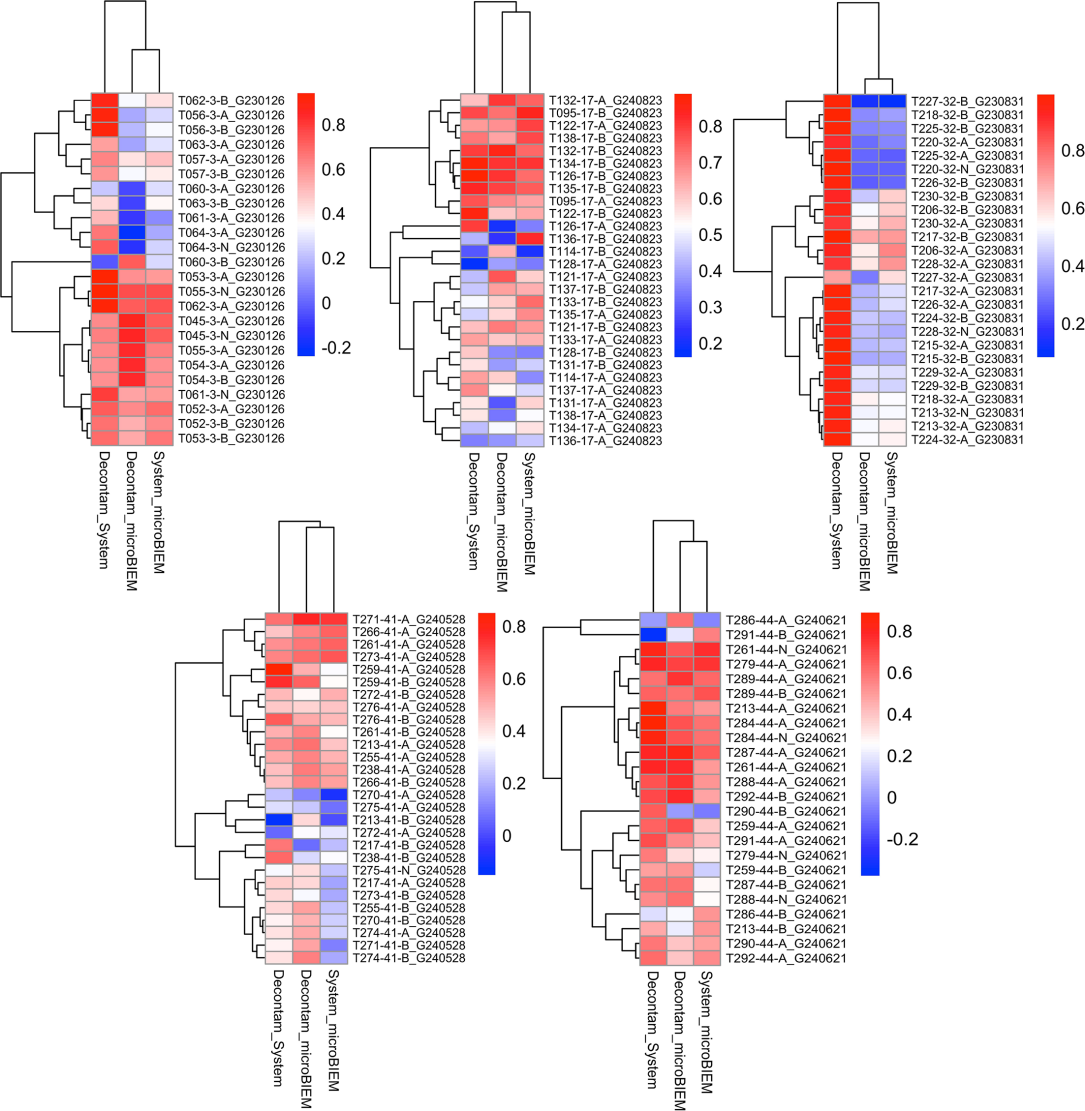
Pearson correlation of the microbial ecology distribution in the samples. The metric used was the JSD index for comparing between the decontamination methodologies ‘Stop-Check-Go’ (system), MicrobIEM and Decontam.

The ‘Stop-Check-Go’ system was compared with Decontam and MicrobIEM methods in the decontamination of ‘tax_ids’ per week, and the correlation between the (dis)similarity of the microbial communities (Fig. 6). A Pearson correlation below 0.3, indicating a high level of dissimilarity between the microbial community structures, was further evaluated. We found a relative decontamination agreement of 66.67% and 75.56% between the ‘Stop-Check-Go’ (System) and Decontam and MicrobIEM, respectively. However, the ‘tax_ids’ that were not decontaminated by Decontam and MicrobIEM had low levels of agreement (14.29% and 4.17%, respectively). The comparison of the Decontam_System and MicrobIEM_System is outlined in Fig. S5 A, B, respectively. We identified that the major limitation of the methods was the decontamination of MNPs. We quantified that MNPs falsely not decontaminated by Decontam represented 22.45% of the dataset (*n*=11/49, Supplementary Material 2 Fig. S5 A). Likewise, MNP not decontaminated by MicrobIEM represented 58.33% of the samples (*n*=14/24, Fig. S5 B). The prevalence inspection of positive samples and negative controls by the Decontam function IsContam with a threshold of 0.1 (default) theoretically splits true from the false contaminants (Supplementary Material 2 Fig. S6 A). However, this inspection in our dataset (after removing MNP and weeks with suspected cross-contamination) showed limitations in differentiating true samples from contaminants even with stricter thresholds like 0.5 (Supplementary Material 2 Fig. S6 B). Likewise, we present a direct comparison of the IsContam (Supplementary Material 2 Fig. S7A) and isNotContam (Supplementary Material 2 Fig. S7B) functions for week 17, which exhibited major limitations in classifying contaminants.

For the assessment of the decontamination by the ‘Stop-Check-Go’ system, we compared how similar the samples were before and after decontamination using ordination-based analyses. This taxonomic composition is shown in the PCoA analysis in Supplementary Material 2 Fig. S8, where raw datasets are represented in red. MNPs cluster with the negative controls, indicating a consistent removal of background contaminants, whereas twins and patients with similar metagenomic profiles also group together in the coordinate space. The details of twins and triplets are provided in Supplementary Material 1 Table S5. The magnitudes of sample change in the ordination space are presented in Supplementary Material 2 Fig. S9: greater distances represent stronger contamination signals that were removed during the decontamination process.

## Discussion

Contaminant species are always present in metagenomic studies, usually introduced from reagent kits, environmental sources and cross-contamination when samples are routinely handled before sequencing. This study addresses the challenge of microbial contamination in long-read metagenomic datasets derived from neonatal nasal and rectal swabs. We developed a comprehensive workflow encompassing pre-sequencing, sequencing and post-sequencing stages to detect putative contaminants and exclude putative human and microbial contaminant DNA. We introduced the ‘Stop-Check-Go’ system that aims to mitigate the introduction of contaminants in downstream analyses using species prevalence, coverage and microbiological reports to refine the identification of microbial taxa with biological meaning. Therefore, we complement our metagenomic analyses with short-read sequencing (Illumina) to validate the presence of Enterobacterales (which we survey) and confirm with better precision the cross-contaminant species in the controls.

Addressing contamination has gained relevance due to the increase of metagenomic study applications, and the use of reproducible decontamination tools is an emerging need. Previous studies in host-associated metagenomes, such as human or mouse samples, have found hDNA contamination to be the most prevalent; for example, 72% of the metagenomes in one study had at least one potential human contamination [38]. In this study, hDNA was an important source of contamination, and the lysis protocol helped to remove almost 87% of hDNA reads; however, the set of samples used to calculate the effectiveness of the lysis method was small. We observed that the removal of hDNA contamination was effective in most cases, but we are aware of the high variability in the results, where the effectiveness can be as low as 70% (Supplementary Material 1 Table S3, sheet: three_conditions_tested). The inclusion of adaptive sequencing (Readfish) targeting the hDNA (during sequencing) and further removal with minimap2 using the T2T-CHM13v2 reference (post-sequencing) will remove the remnant host DNA.

To date, there are 51 different tools for removing host contamination [36,39, 40], and the microbial decontamination tools vary depending on the compositionality of the dataset, the sequencing technology and the specific needs of the metagenomic studies. However, most decontamination tools are designed and tested in short-read metagenomic microbiomes [7,10, 41,43], which pose an evident limitation for use in our dataset. In addition, the identification of contaminants in low biomass samples adds a layer of difficulty, where the tools have been published with a limited detection capacity when separating controls from true samples [44].

In our present study, we aim to emphasize the use of negative controls to mitigate putative contaminants through the detection of common shared species per sequencing run. Previous studies reported costs and time as limitations [44]. We calculated that using two negative controls represented, in our study, 0.78% (<1%) of the sequencing costs (calculation based on one flow cell, demultiplexed with 15 samples). In the absence of negative controls, some decontamination tools use a list of common species reported as contaminants from extraction kits and environmental sources [9,44,46], under the assumption that reads matching these external references are considered contaminants. We showed changes weekly in the taxonomic profiles relating to things like lot changes; hence, in agreement with Salter *et al*. [3], we advise against comparing with external controls or sources.

As sequencing technologies continue to evolve, the ability to effectively manage contaminants will remain crucial for producing high-quality data and uncovering meaningful biological insights. The lack of sufficient negative controls to distinguish contaminants from real community species increases the risk of misinterpretation. Metagenomic studies frequently do not carry out microbiological growth controls [47,49], as their samples may include unculturable species. Employing a coverage threshold can be one way of filtering putative microbial contaminants. We considered that the investigation of Enterobacterales, the enrichment step we performed and the inclusion of weekly negative controls were advantages compared with other studies that investigated the abundant fraction of non-culturable microbes.

The three parameters included in the ‘Stop-Check-Go’ classification system differentiate those patients who showed a significant number of bacterial reads as putative contaminants. We argue that using coverage as one of the classification parameters outperforms using read counts as coverage adjusts for read length and genome size. The analysis of species in controls suggests that a proper threshold needs to be established to exclude most misclassified species when read counts are used as the classification parameter. Coverage in our dataset provided reliable classifications as 80% of the species in microbiologically negative samples were excluded, but it is well complemented by the prevalence method [7], and microbiological reports. Using the combination of these three parameters, we could observe in our dataset that for the same week, species can be simultaneously classified as ‘Go’ and as putative contaminants to ‘Stop’ (Fig. 5). The decontamination tools we have tested – Decontam and MicrobIEM – generate a list of putative contaminant ‘tax_ids’ in the dataset that are removed globally. We highlight that the ‘Stop-check-go’ (System) provides a combination of ‘tax_ids’ and ‘sample_ids’, which makes the decontamination of samples species-specific. The decontamination agreement between the System, Decontam and MicrobIEM is on average 71%, represented in red colours in all the weeks (Fig. 6). The largest differences between the decontamination tools were attributed to Decontam and MicrobIEM failing to exclude putative contaminants present in high abundance in MNPs.

The decontamination is also influenced by the complexity of the metagenomic samples. We observed negative controls with a low number of reads and coverage above 10x, characteristic of low-complexity metagenomes where some species are over-represented (most likely library contaminants). In medium- to high-complexity metagenomes, the species will require high-quality reads to end up in good coverages and lower rejection rates. We found the prevalence method robust enough when complemented with other parameters as in our study for the identification of bacterial contaminants.

Decontamination efforts in metagenomic studies are particularly relevant when considering clinical applications, where time-sensitive decisions often need to be made. In many cases, traditional culture methods are performed, but their results become available later. In contrast, metagenomic sequencing can provide rapid insights into the microbial composition of samples. Therefore, minimizing contamination is essential to ensure the reliability of the sequencing data, as clinicians may need to rely on this information to guide early diagnosis and treatment decisions before culture results are available.

As contamination is not completely avoidable, mitigation strategies and resources should be employed. Whilst it is crucial to address microbial contaminants introduced during sample handling or from extraction and library preparation kits, an equally strict removal of hDNA contaminants is a critical step in ensuring the accuracy and reliability of metagenomic studies [38]. We employed strategies to mitigate contamination rather than discarding our data. The first strategy was to perform a lysis protocol before DNA extraction, and the use of Readfish from week 17 after we detected the introduction of contaminants in low biomass samples, reducing the sequenced reads matching the hDNA and increasing the microbial reads. We are not explicitly recommending the use of selective sequencing to accompany the Stop-Check-Go system; however, we consider an hDNA depletion step to be essential before sequencing. A second strategy was to cryopreserve positive Enterobacterales samples, and third, to sequence them with MiSeq (Illumina) to confirm the species and sequence type. Fourth, including weekly negative controls and applying robust computational tools helped to mitigate the rate of false positives and false negatives, especially in low-biomass samples [4]. We implemented nearly 70% of the guidelines published by Fierer *et al*. [6]. Two main strategies were not considered as they were unknown to us prior to the start of this project: first, a spatial arrangement with high-biomass samples distant from low-biomass samples, as this might favour the cross-contamination in the steps prior to sequencing. This would have been difficult to implement, as the level of colonization of patients is unknown at the time of sampling, and all samples of a single week would be processed together. Second, the reagent batch change and the geographical distribution reports of samples are useful for using tools such as SCRuB [11].

We acknowledge limitations in our study. We could not test the effect of additional measures for contamination mitigation, such as the strict disinfection of equipment and changing DNA extraction kits, because the final volume of the samples was less than 700 µl and only processes after DNA extraction could be repeated. Also, the samples negative for Enterobacterales from microbiology reports were not cryopreserved, and if the reports from metagenomic sequencing disagreed, it was not possible to recover the sample. We assume that the repeated libraries have the best library quality given the lower error rate, the improvement in performance of R10 chemistry and a rise in the loading volume (initial concentration), with the exception of weeks 25, 38 and 39, from which we used the original library as the repetition did not increase the library concentration, and there was no significant improvement in the controls’ taxonomic profile (Fig. 3).

## Conclusions

The parameters described in this study included laboratory and bioinformatics work and did not model species contamination. We aimed to mitigate the introduction of contaminants and to keep species with biological significance that can be falsely excluded by the decontamination tools tested. The validation with microbiological growth reports, the prevalence method [7] and coverage from assemblies using FLYE and Kraken-translate were key in assessing contaminant species. We are aware that the metagenomes in this study did not represent the actual microbiome of the patients due to the enrichment step but served to increase the microbial ratio in our low-biomass samples from neonates. The advantage of the conceptualization of the ‘Stop-Check-Go’ system is that we could individually examine the species classified as ‘Check’ in the context of tracking Enterobacterales. We are also aware that examining individual cases may involve a trade-off with the desired high-throughput processing. However, it is well recognized that metagenome analysis poses a significant challenge due to the complexity of the samples, where precision and biological relevance should take precedence over speed.

## Supplementary material

10.1099/mgen.0.001609 Supplementary Material 1.

10.1099/mgen.0.001609 Supplementary Material 2.

## Abbreviations

BHI: brain heart infusion
DFR: difference fraction reads
hDNA: human DNA
JSD: Jensen–Shannon diversity
1K: 1,000
10K: 10,000
MNPs: microbiologically negative patients
NICU: neonatal intensive care unit
ONT: Oxford nanopore technology
ST: sequence type
TAPIR: tracking the acquisition of pathogens in real-time
