# Supplementary material for: Mitigation and detection of putative microbial contaminant reads from long-read metagenomic datasets
Source: Microb Genom. 2026 Jan 22;12(1):001609. doi: 10.1099/mgen.0.001609 (PMC12828179; doi:10.1099/mgen.0.001609)
Supplement: Supplementary Material 1. [file mgen-12-01609-s001.pdf]

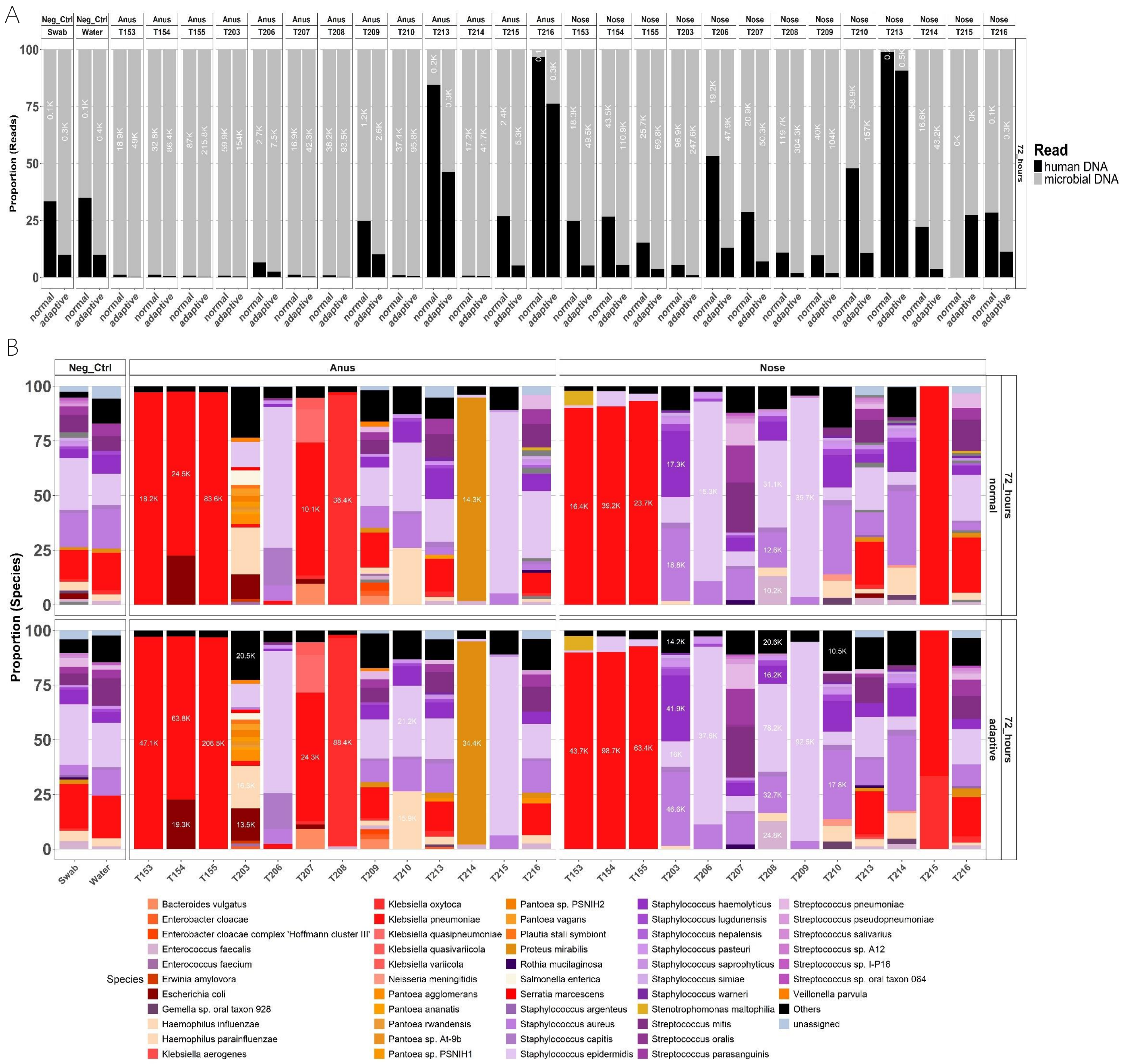

Supplementary Figure S1. Comparison of adaptive and normal sequencing using Readfish. A. Number of reads in week 30 at 72 hours of sequencing. B. Proportion of the species classified with Bracken at 72h using normal and adaptive sequencing in the same week (week 30).

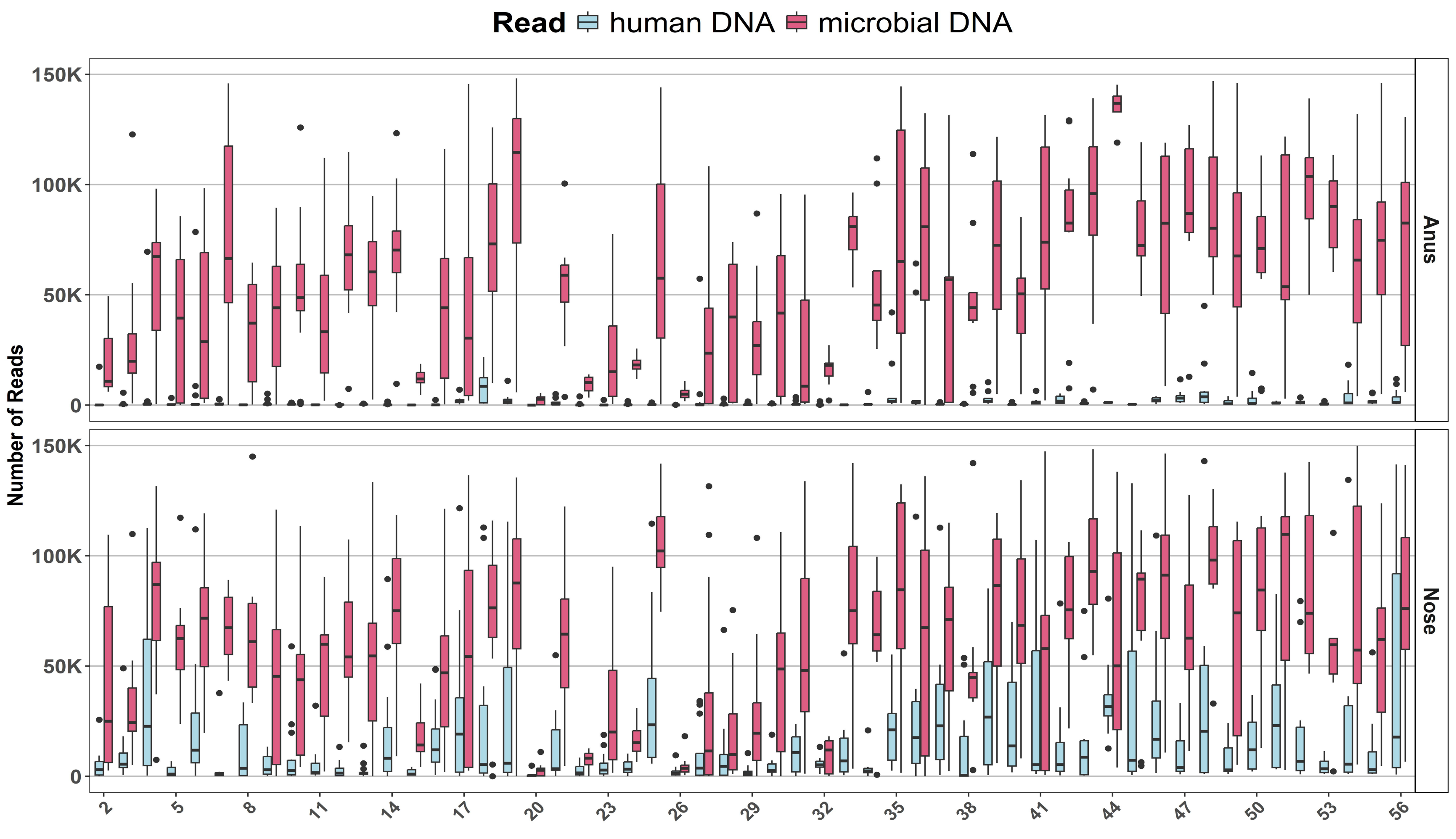

Supplementary Figure S2. Number of reads in human and microbial DNA. The reads are chronologically ordered for the sources sampled: anus and nose.

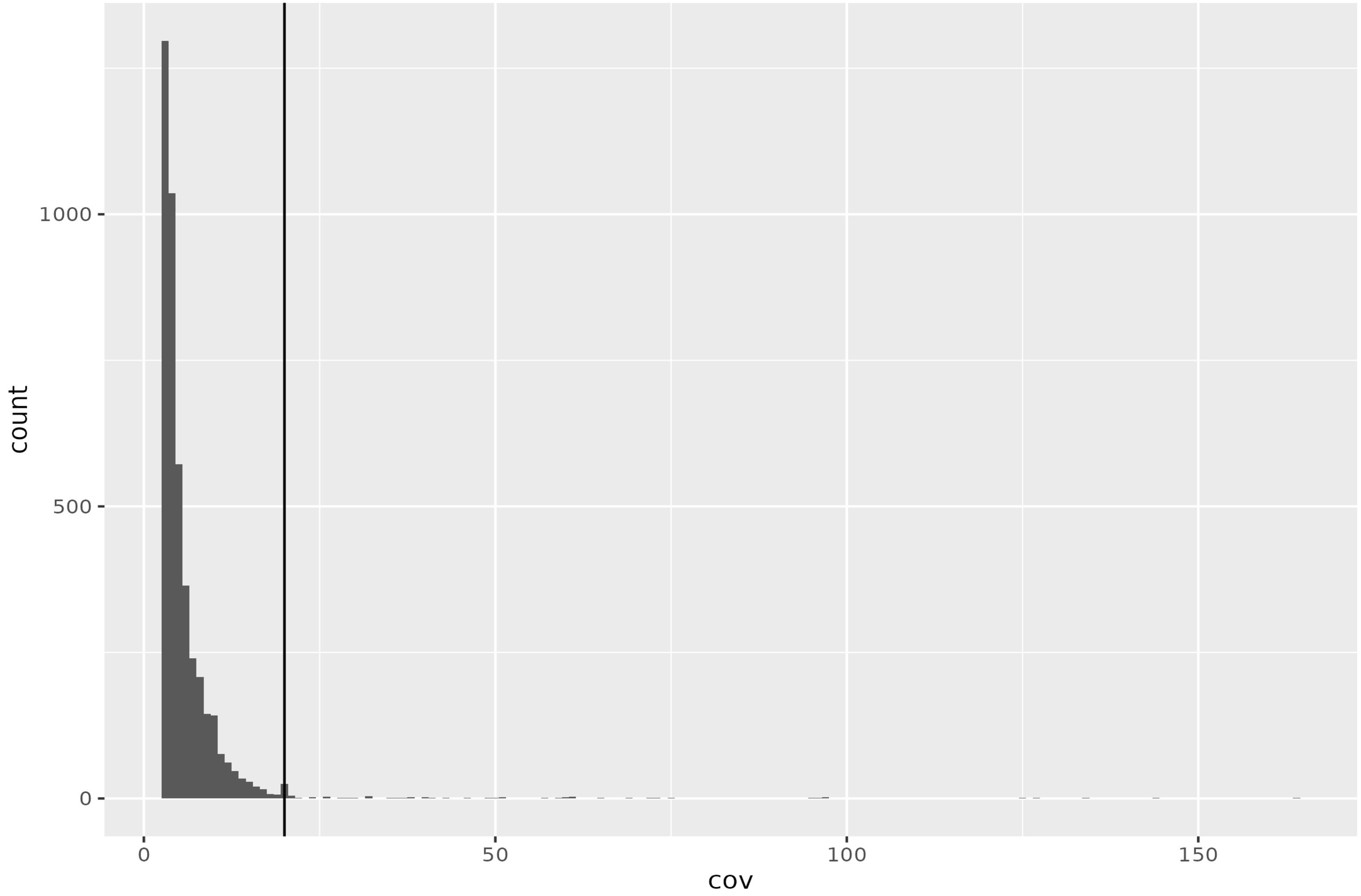

Supplementary Figure S3. Distribution of the coverage for the negative controls and microbiologically negative samples. The vertical line refers to the chosen threshold of 20X.

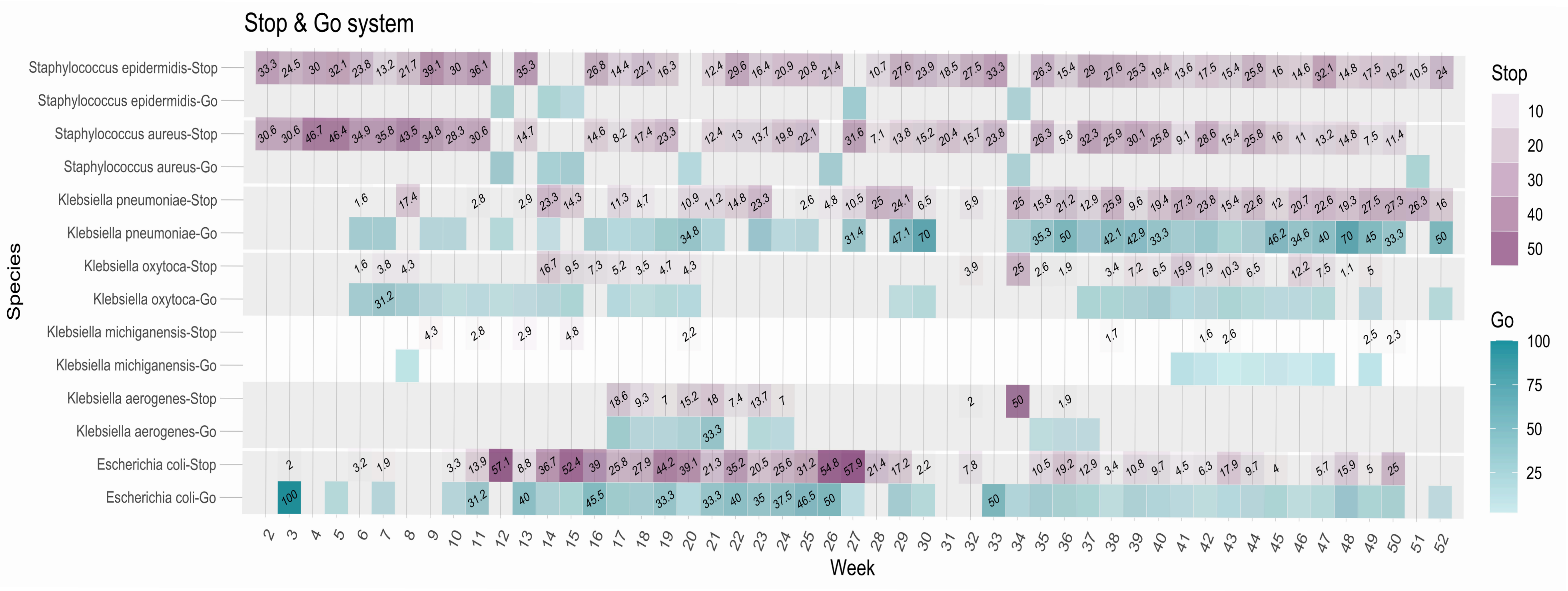

Supplementary Figure S4. The most abundant species in the dataset classified as 'Stop' and 'Go' in the system. The relative abundances of the bacterial species are labelled, except values below 10%.

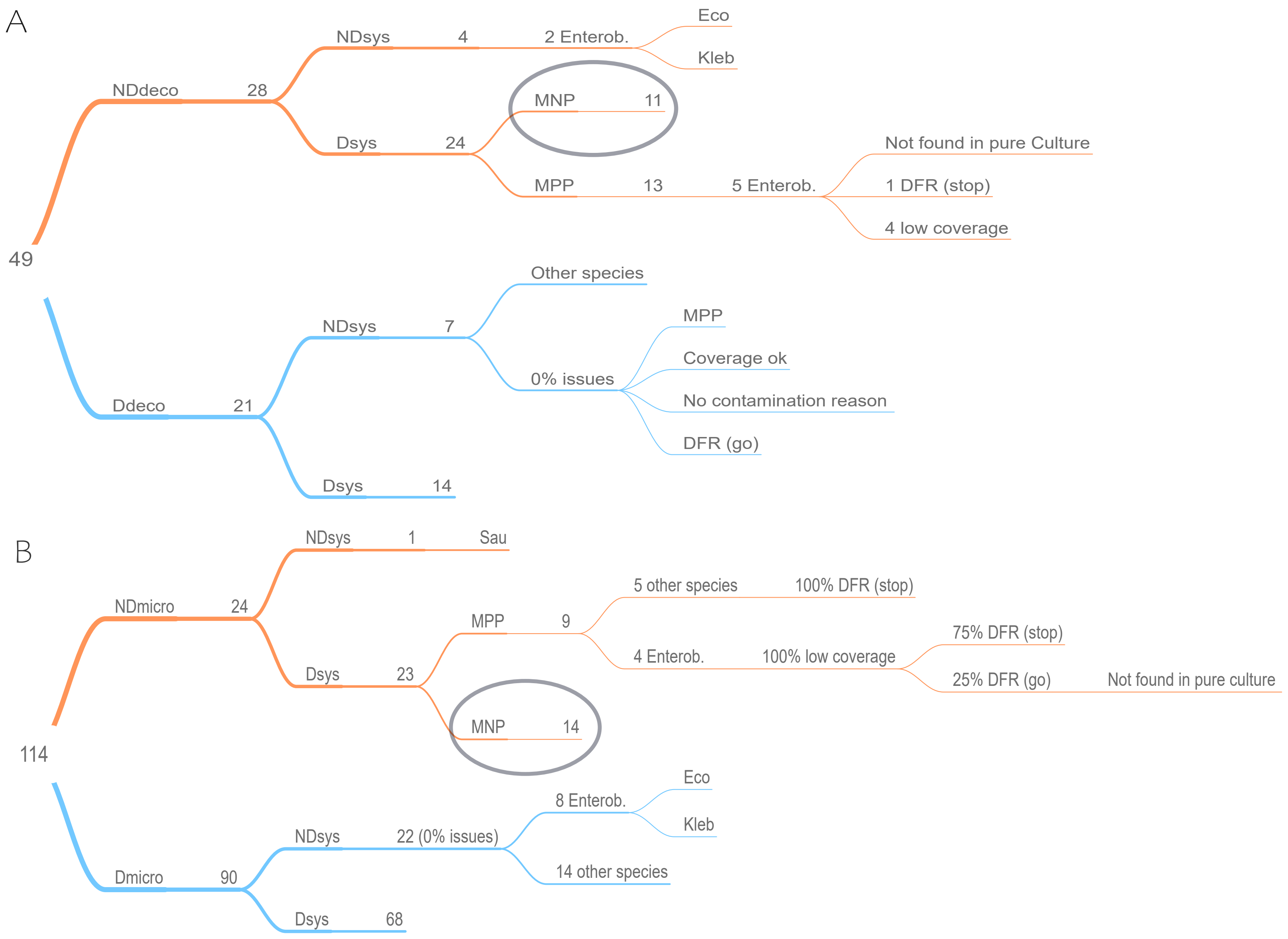

Supplementary Figure S5. Tree diagram comparing the results for the paired decontamination tools tested. ND refers to 'not decontaminated', decontaminated (D), A. Decontam (deco), 'Stop-Check-Go' (sys) B. MicroBIEM (micro) 'Stop-Check-Go' (sys).

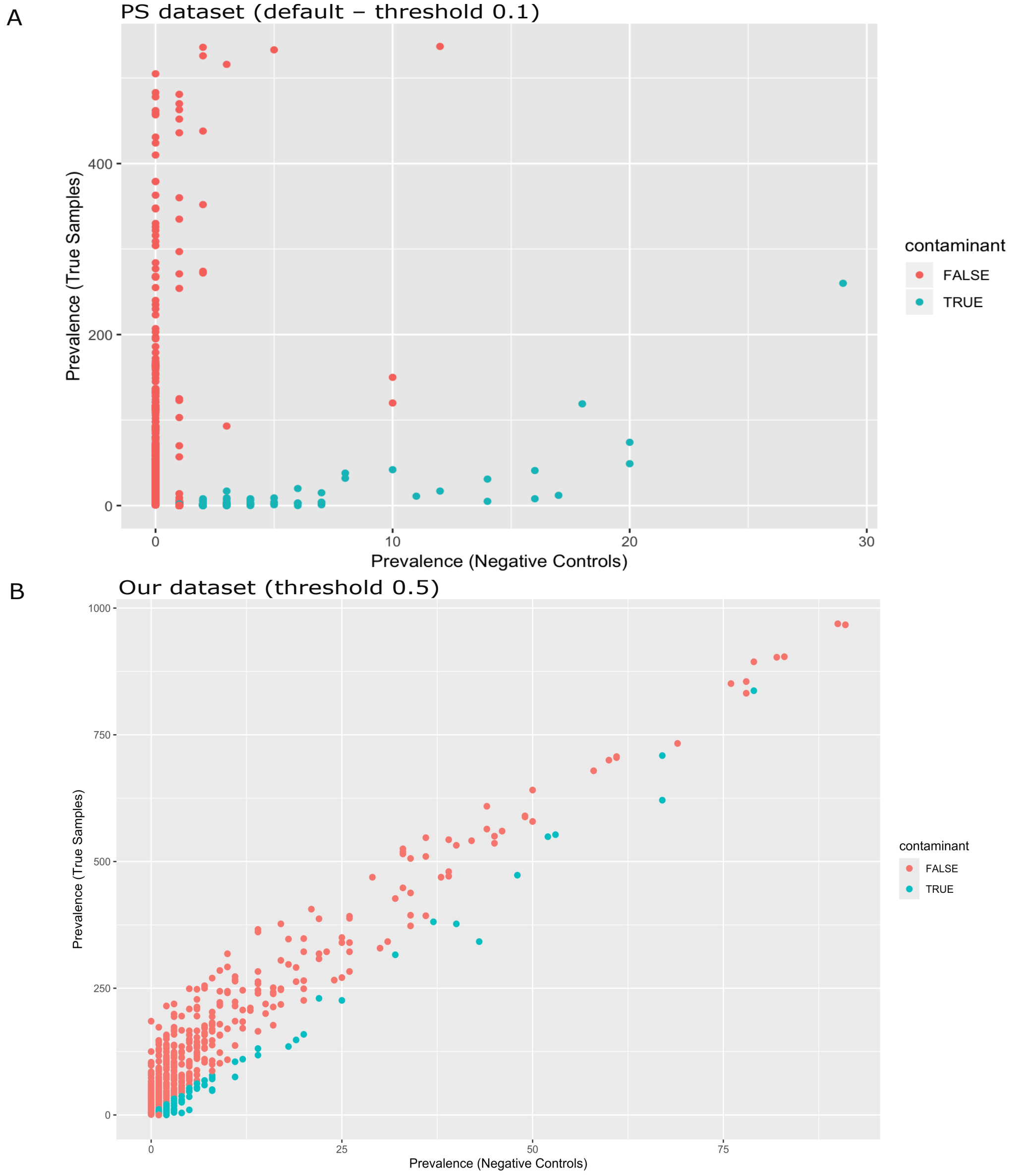

*Weeks with suspected cross-contamination were excluded (2, 4, 7, 12, 13, 14, 15, 16, 20, 26, 29, 48)

Supplementary Figure S6. Result of the prevalence method application (only) from Decontam. A. PS dataset (published in https://benjjneb.github.io/decontam/vignettes/decontam_intro.html, section 7) with the default threshold (0.1). B. In our dataset, excluding microbiologically negative patients (MNP) and weeks with suspected cross-contamination (with a stricter threshold of 0.5).

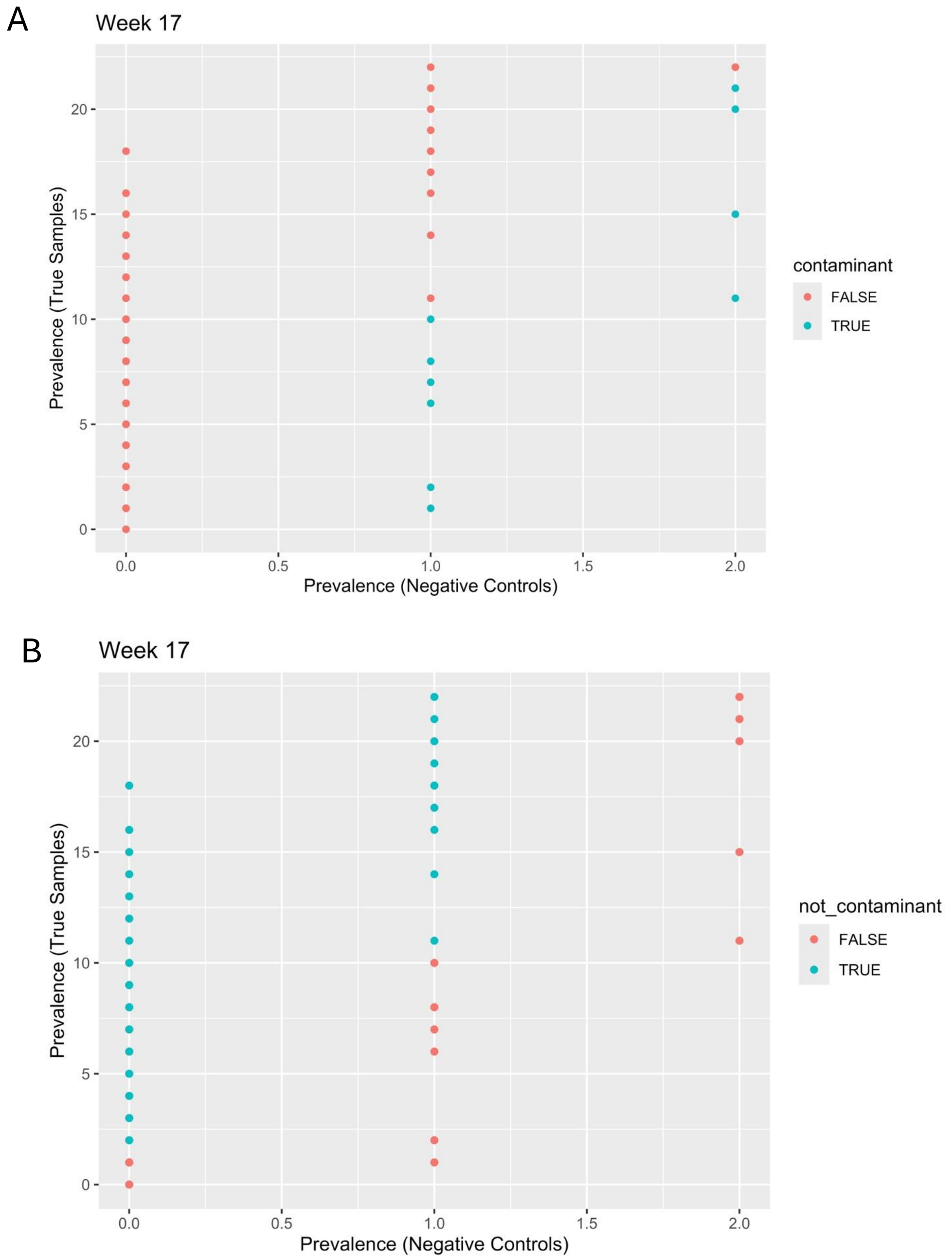

Supplementary Figure S7. Comparison of the prevalence method of Decontam R package on week 17. The functions used are A. *IsContam*, B**.** *IsNotContam***.**

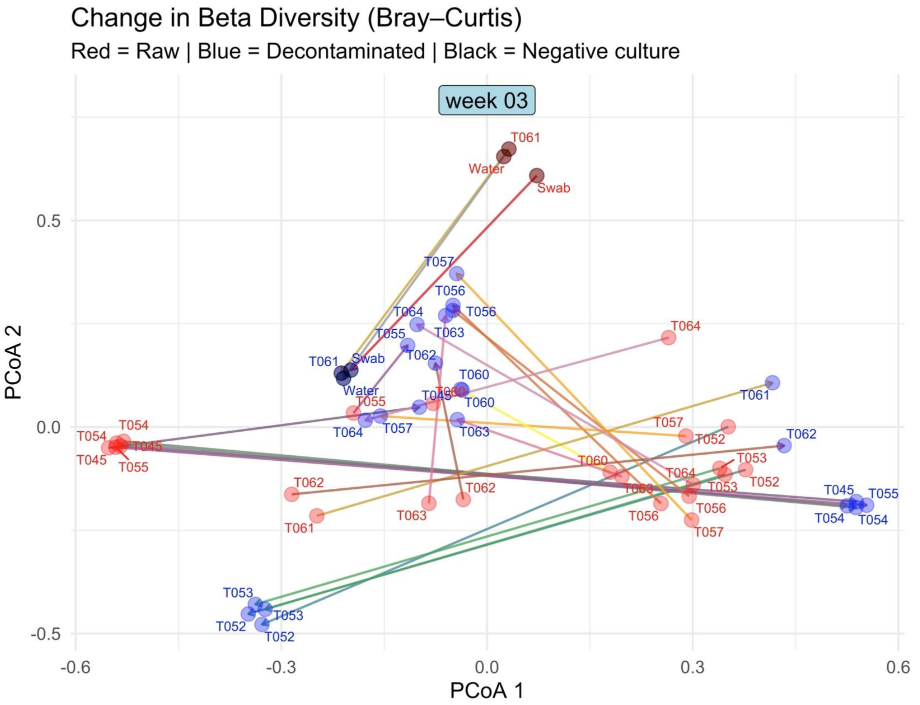
Change in Beta Diversity (Bray–Curtis)
Red = Raw | Blue = Decontaminated | Black = Negative culture
week 03
PCoA 1
PCoA 2
-0.6
-0.3
0.0
0.3
0.6
-0.5
0.0
0.5

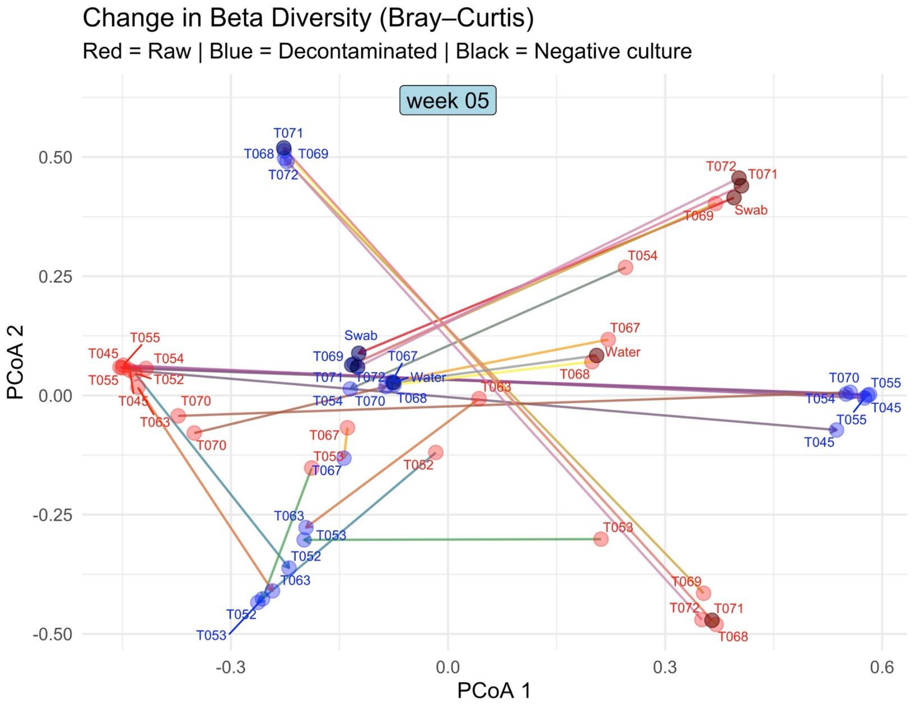
Change in Beta Diversity (Bray–Curtis)
Red = Raw | Blue = Decontaminated | Black = Negative culture
week 05
PCoA 1
PCoA 2
-0.3
0.0
0.3
0.6
-0.50
-0.25
0.00
0.25
0.50

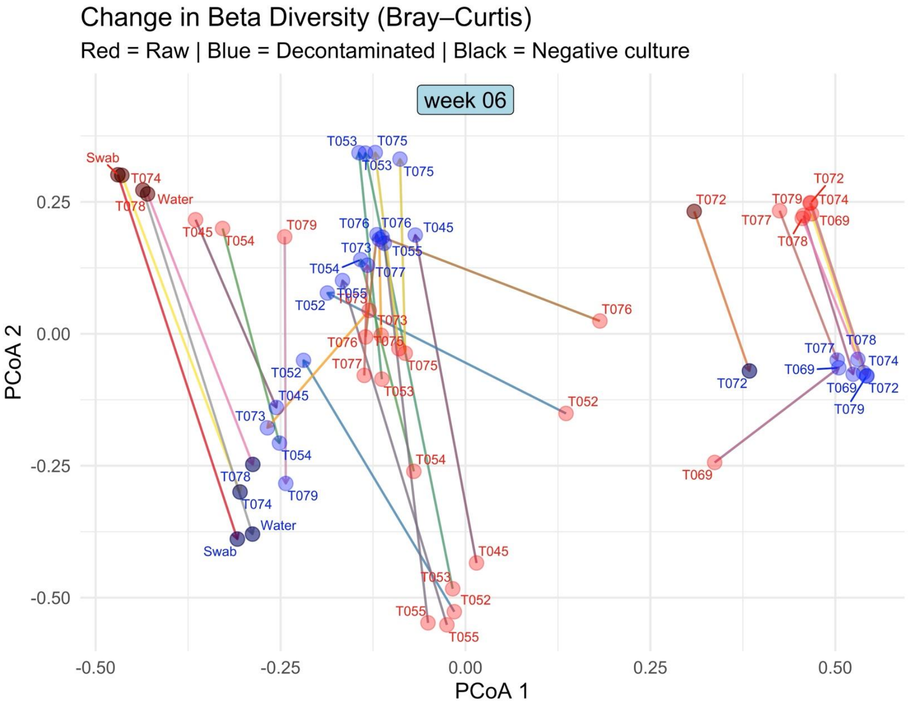
Change in Beta Diversity (Bray–Curtis)
Red = Raw | Blue = Decontaminated | Black = Negative culture
week 06
PCoA 1
PCoA 2
-0.50
-0.25
0.00
0.25
0.50
-0.50
-0.25
0.00
0.25

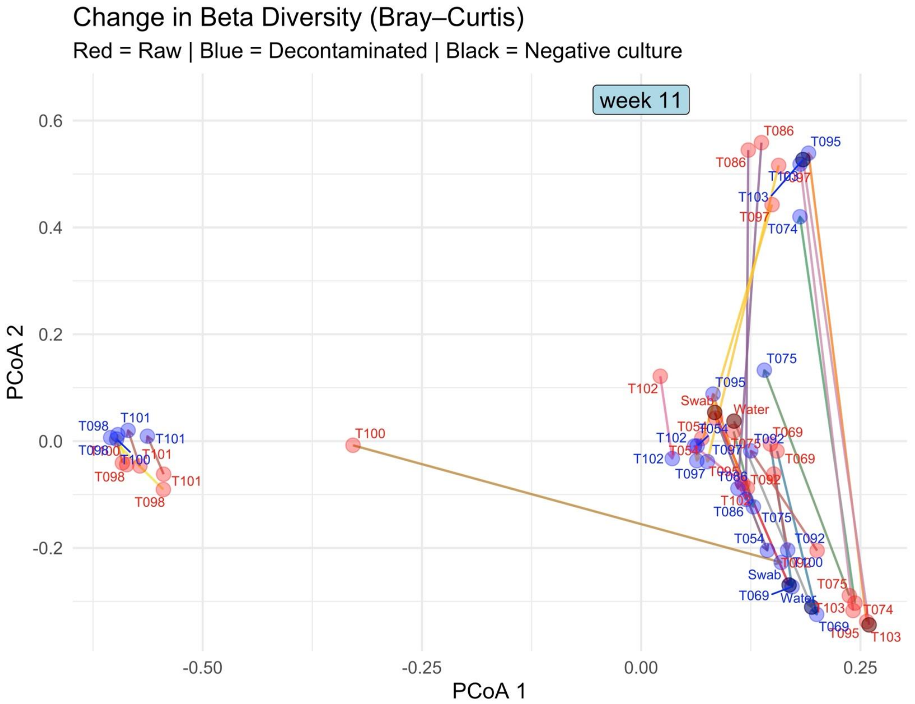
Change in Beta Diversity (Bray–Curtis)
Red = Raw | Blue = Decontaminated | Black = Negative culture
week 11
PCoA 1
PCoA 2
-0.50
-0.25
0.00
0.25
-0.2
0.0
0.2
0.4
0.6

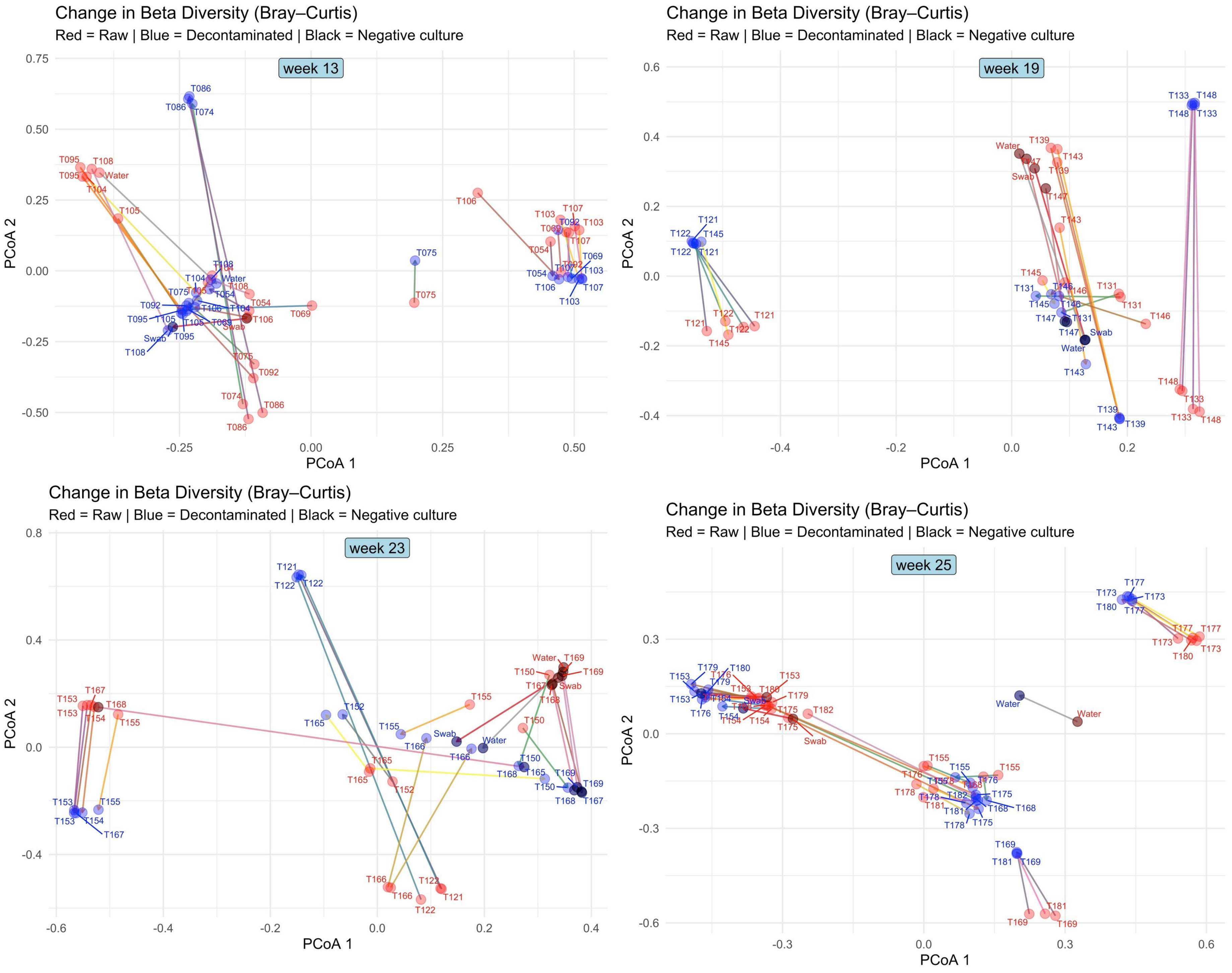
Change in Beta Diversity (Bray–Curtis)
Red = Raw | Blue = Decontaminated | Black = Negative culture
week 13
PCoA 1
PCoA 2
0.75
0.50
0.25
0.00
-0.25
-0.50
-0.25
0.00
0.25
0.50
Change in Beta Diversity (Bray–Curtis)
Red = Raw | Blue = Decontaminated | Black = Negative culture
week 19
PCoA 1
PCoA 2
0.6
0.4
0.2
0.0
-0.2
-0.4
-0.4
-0.2
0.0
0.2
Change in Beta Diversity (Bray–Curtis)
Red = Raw | Blue = Decontaminated | Black = Negative culture
week 23
PCoA 1
PCoA 2
0.8
0.4
0.0
-0.4
-0.6
-0.4
-0.2
0.0
0.2
0.4
Change in Beta Diversity (Bray–Curtis)
Red = Raw | Blue = Decontaminated | Black = Negative culture
week 25
PCoA 1
PCoA 2
0.3
0.0
-0.3
-0.6
-0.3
0.0
0.3
0.6

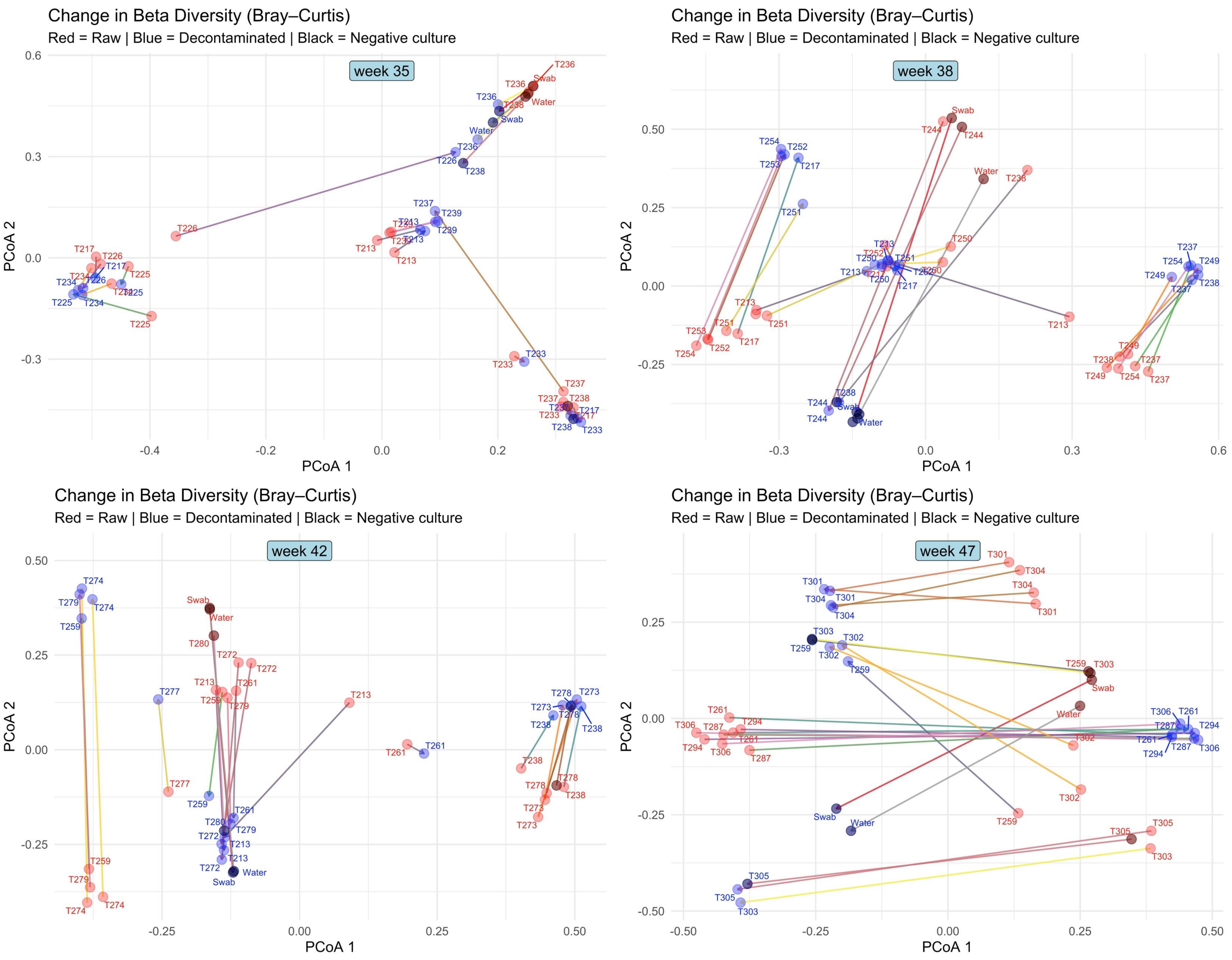
Change in Beta Diversity (Bray–Curtis)
Red = Raw | Blue = Decontaminated | Black = Negative culture
week 35
PCoA 1
PCoA 2
Change in Beta Diversity (Bray–Curtis)
Red = Raw | Blue = Decontaminated | Black = Negative culture
week 38
PCoA 1
PCoA 2
Change in Beta Diversity (Bray–Curtis)
Red = Raw | Blue = Decontaminated | Black = Negative culture
week 42
PCoA 1
PCoA 2
Change in Beta Diversity (Bray–Curtis)
Red = Raw | Blue = Decontaminated | Black = Negative culture
week 47
PCoA 1
PCoA 2

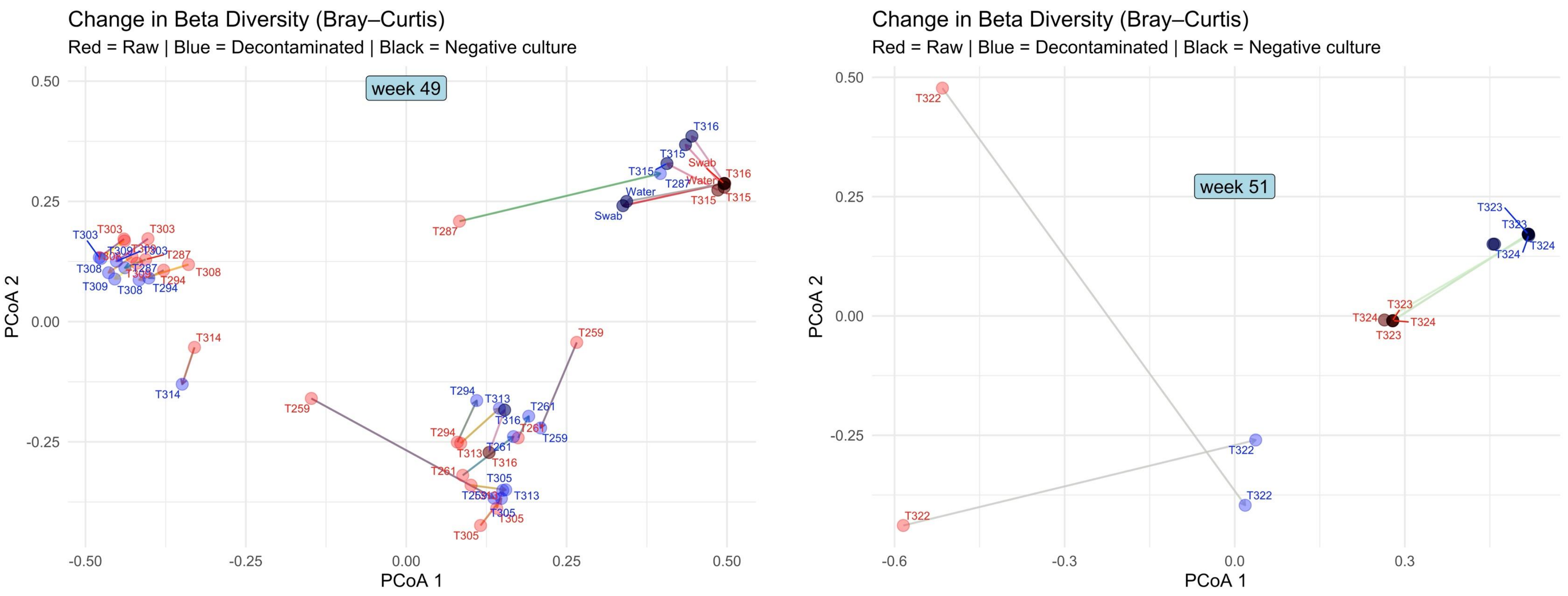

Supplementary Figure S8. PCoA distribution of samples using Bray-Curtis distance for the biom files published in https://github.com/SAM81221/Stop-Check-Go_TAPIR. Negative culture samples are highlighted in black, raw dataset in red, and decontaminated by 'Stop-Check-Go' system in blue. During week 51 (18 – 24 December, we received only 6 samples from the ward (3 patients).

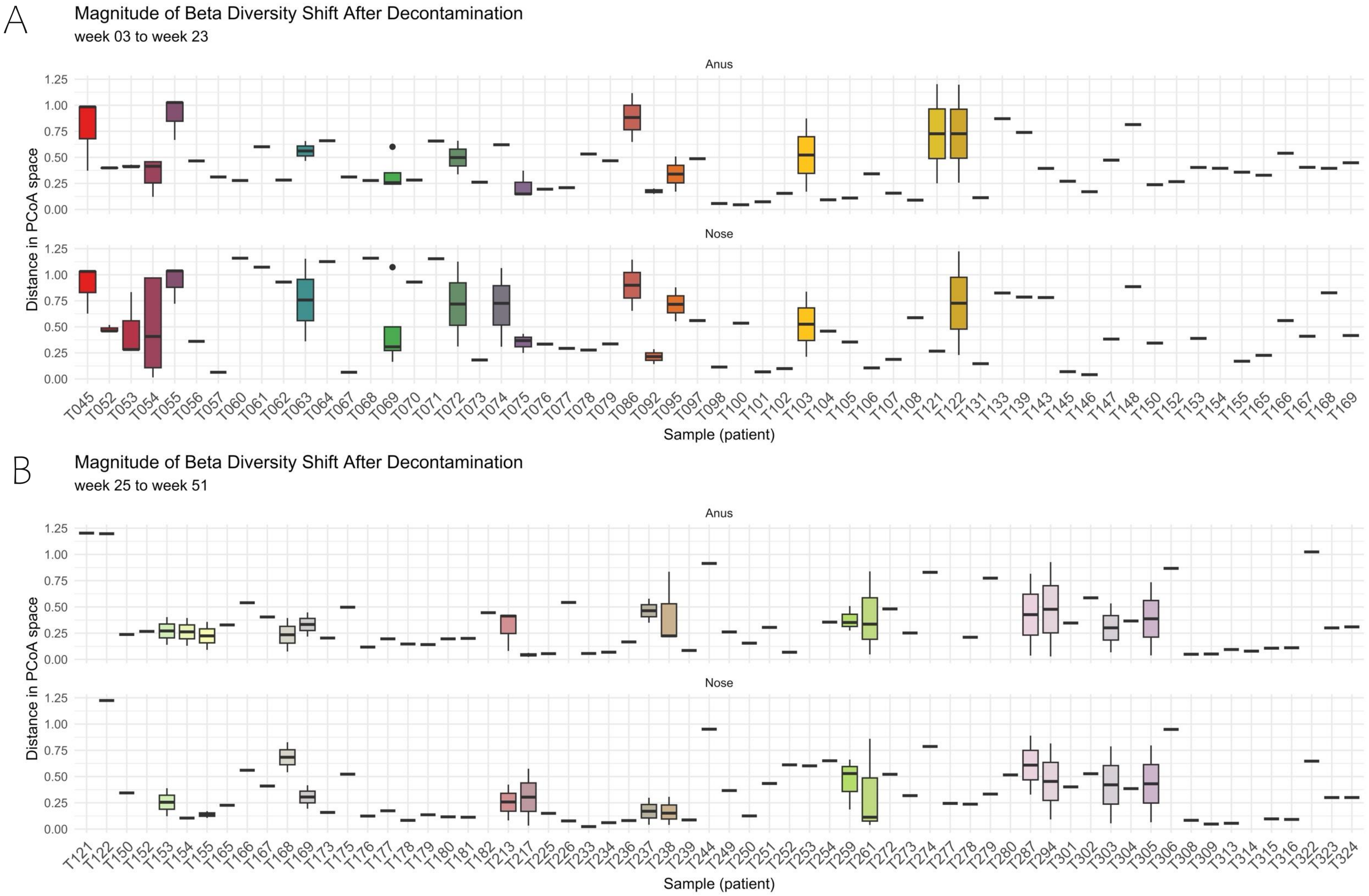

Supplementary Figure S9. Magnitude of the shift in the PCoA space for the biom files published in https://github.com/SAM81221/Stop-Check-Go_TAPIR. Comparison between the raw and the decontaminated samples split by source (anus, nose) from A. Week 03 to week 23, and B. Week 25 to week 51.
